# A network meta-analysis of the efficacy of hypoxia-inducible factor prolyl-hydroxylase inhibitors in dialysis chronic kidney disease

**DOI:** 10.18632/aging.204611

**Published:** 2023-03-27

**Authors:** Jun Chen, Xinyang Shou, Yanyan Xu, Lie Jin, Chaoyong Zhu, Xiaolan Ye, Ziwei Mei, Peipei Chen

**Affiliations:** 1 Zhejiang Chinese Medical University, First Clinical Medical College, Hangzhou, Zhejiang 310000, China; 2 Department of Pharmaceutical, Lishui Municipal Central Hospital, The Fifth Affiliated Hospital of Wenzhou Medical University, Lishui, Zhejiang 323000, China; 3 Department of Nephrology, Lishui Municipal Central Hospital, The Fifth Affiliated Hospital of Wenzhou Medical University, Lishui, Zhejiang 323000, China; 4 Department of Pharmaceutical, Zhejiang Provincial People’s Hospital, Hangzhou, Zhejiang 310000, China

**Keywords:** hypoxia-inducible factor prolyl-hydroxylase inhibitors, erythropoiesis-stimulating agents, anemia, chronic kidney disease, dialysis

## Abstract

Background: Five types of HIF-PHIs have been authorized for anemia treatment in CKD patients in China and Japan. These are enarodustat, roxadustat, daprodustat, vadadustat, and molidustat. How effectively they compare to ESAs about clinical results in CKD-DD patients is uncertain. This study examined the RCT evidence about the benefits and risks of HIF-PHIs and ESAs in dialysis CKD patients.

Methods: We conducted an extensive investigation and network meta-analysis of RCTs. In these RCTs, patients with CKD-DD received one of five different HIF-PHI or ESAs, a placebo, and no medical intervention. Outcomes included hemoglobin, iron parameters, and adverse events, and there were four weeks of follow-up at least. A frequentist framework for multivariate random effects meta-analyzed the results. The effect sizes of categorical variables were displayed as odds ratios. Mean differences were employed for computing continuous outcomes with common units; otherwise, standardized mean differences were applied. The Cochrane tool evaluated the bias risk in RCTs.

Results: 26 RCTs with 14945 patients were qualified for inclusion. Compared to the placebo, HIF-PHIs and ESAs dramatically boosted hemoglobin without affecting serum iron. Roxadustat performed better hemoglobin levels than ESAs (MD 0.32, 95% CI 0.10 to 0.53) and daprodustat (0.46, 0.09 to 0.84). Roxadustat (91.8%) was the top hemoglobin treatment among all medical interventions, as determined by the SUCRA ranking. However, roxadustat caused more thrombosis and hypertension than ESAs (1.61, 1.22 to 2.12) and vadadustat (1.36, 1.01 to 1.82). The lowest rates of hypertension and thrombosis were seen in molidustat (80.7%) and ESAs (88.5%). Compared with a placebo, ESAs and HIF-PHIs all affected TSAT levels. Except for molidustat, the other four HIF-PHIs impact different iron parameters. Regarding ferritin reduction, roxadustat (90.9%) and daprodustat (60.9%) came out on top. Enarodustat (80.9%) and roxadustat (74%) placed best and second in lowering hepcidin levels. The former two medicines for TIBC improvement were vadadustat (98.7%) and enarodustat (80.9%).

Conclusion: The most effective treatment for hemoglobin correction is roxadustat. The superior efficacy of reducing hepcidin makes roxadustat and enarodustat appropriate for patients with inflammation. However, the increased risk of hypertension and thrombosis associated with roxadustat should be noted. In patients at risk for hypertension and thrombosis, molidustat and ESAs may be preferable options. When administering roxadustat and daprodustat, clinicians should check ferritin to assess iron storage. Lower TSAT in patients receiving HIF-PHIs and ESAs treatment suggests intravenous iron supplements are needed.

## INTRODUCTION

Renal anemia is a prevalent and serious complication among CKD patients. It is common in many nations and linked to poor clinical outcomes, including hospitalization, declining health-related quality of life, and mortality [1, 2]. Anemia was defined as a hemoglobin level less than 130 g/L for males or 120 g/L for females over 15 years old in the clinical practice guideline of KDIGO in 2012 [3]. Renal anemia is caused by reduced red blood cell (RBC) survival and erythropoietin (EPO) production due to renal failure [4–7]. Furthermore, iron availability is necessary for erythropoiesis. Hepcidin and ferroportin contribute to the iron balance in humans. Elevated hepcidin and decreased ferroportin result in an iron shortage and sequestration, essential for renal anemia [8].

RBC transfusions and medicinal treatments, such as ESAs and HIF-PHIs, treat renal anemia in CKD patients. ESAs encourage erythropoiesis and lessen the requirement for RBC transfusions. KDIGO suggested that ESAs should treat CKD-DD patients with 9–10 g/dL to prevent a hemoglobin level of less than nine g/dL [1, 9]. However, ESAs are administrated subcutaneously or intravenously. In the long term, they most likely raise the risk of thrombosis and hypertension [10, 11]. Moreover, some patients are resistant and hypo-response to ESAs [12, 13]. Several factors are related to this hypo-responsiveness, including inflammation, nutritional state, and dialysis adequacy. Some people require a larger dose than usual to obtain the desired hemoglobin concentration. A higher dose of ESAs increases morbidity and death invariably [14, 15].

Unlike ESAs, HIF-PIHs are oral medications. They inhibit PHI to enhance HIF-mediated endogenous EPO synthesis. Furthermore, HIF-PHIs decrease hepcidin levels to improve iron availability. In 2020, the Asian Pacific Society of Nephrology (APSN) indicated that HIF-PHIs might be used as an alternative to ESAs in the correction and maintenance of hemoglobin for CKD patients receiving dialysis or not [16]. Nowadays, five kinds of HIF-PHIs have been approved in China and Japan for anemia treatment in CKD patients [17–21]. They are roxadustat, daprodustat, vadadustat, molidustat, and enarodustat. However, there is a lack of information regarding the relative advantages of HIF-PHIs and ESAs in CKD-DD patients in clinical guidelines and reported trials. The APSN questions their blood pressure and thrombosis safety. These unknowns create uncertainty in selecting an agent from among the five ESAs and HIF-PHIs.

The vast majority of reported meta-analyses compared roxadustat and daprodustat to a placebo. Only one network meta-analysis examined the distinction between HIF-PHIs and ESAs or placebo in CKD-NDD patients [22]. Due to a difference in iron influence among five HIF-PHIs and ESAs and clearance rate of HIF-PHIs between CKD-DD and CKD-NDD patients, we conducted this network meta-analysis to compare the clinical efficacy and safety of ESAs and five HIF-PHIs in patients with CKD-DD.

## METHODS

We registered the study protocol at the International Prospective Register of Systematic Reviews (CRD42022313670). We conducted the study according to the Preferred Reporting Items for Systematic Reviews and Meta-Analyses (PRISMA) guidelines.

### Eligibility criteria

Trials were eligible for inclusion if they (i) were RCTs; (ii) included participants over the age of 18 years with CKD-DD; (iii) compared five kinds of HIF-PHIs with placebo, ESAs, or no medical interventions; (iv) reported any of the following outcomes: differences in hemoglobin levels; differences in iron parameters (ferritin, hepcidin, TIBC, TSAT, serum iron); adverse cardiovascular events (hypertension, thrombosis). Studies covering pediatric patients and patients with CKD-NDD were excluded.

HIF-PHIs therapy in eligible RCTs included roxadustat, daprodustat, vadadustat, molidustat, and enarodustat. ESAs covered epoetin and darbepoetin. If a study contained two or more groups of the same experimental intervention, we combined data from these groups.

### Data sources and searches

Two independent blind reviewers (MZW and CJ) searched PubMed, Embase, Cochrane, Web of Science, and https://clinicaltrials.gov databases from inception to July 7, 2022. A third reviewer (JL) resolved disagreements. The search terms were roxadustat, daprodustat, molidustat, vadadusta, enarodustat, epoetin, darbepoetin, erythropoiesis-stimulating agent, chronic kidney disease, and dialysis. Additional studies were searched in the reference lists and relevant systematic reviews and meta-analyses.

### Data extraction

Two reviewers (CJ and XYY) independently evaluated the eligibility of titles, abstracts, and full texts. Two reviewers (LSM and CPP) independently extracted data from eligible studies. Data included study characteristics (author, publication year), population (sample size, patient demographics, comorbidities), intervention description (drug name, dose), and outcomes. Reviewers resolved disagreements by discussion or consultation with a third reviewer (ZCY).

The primary outcome was hemoglobin change from baseline to end-up. Secondary outcomes were changes in ferritin, hepcidin, TIBC, TSAT, serum iron, and cardiovascular adverse events from baseline to end-up. Cardiovascular adverse events were hypertension and thrombosis.

### Risk of bias assessment

The Cochrane tool independently assessed the bias risk of eligible RCTs by three reviewers (CJ, MZW, and YXL). This tool included six items: random sequence generation, allocation concealment, blinding, incomplete outcome data, selective reporting, and any other bias. In evaluating every study, investigators judged the low, unclear, or high risk of bias for each item and assigned an overall risk of bias.

### Data analysis

For continuous outcomes, outcomes with the same unit were calculated as mean differences (MD) with 95% CI. Standardized mean differences (SMD) with 95% CI were calculated when continuous outcomes varied in measurement units. The results were synthesized as odds ratios (OR) with 95% CI for dichotomous outcomes.

We developed a network meta-analysis by a frequentist method. This method restricts maximum likelihood estimation to quantify network heterogeneity and account for the correlations between effect sizes in studies with more than two groups. Network consistency and common heterogeneity across all treatment contrasts were analyzed. We presented a summary effect of all comparisons by forest plots. Mean ranking and Surface Under the Cumulative Ranking curve (SUCRA) were computed to obtain treatment hierarchies. SUCRA determines the probability of a treatment being the most effective. The larger surface area under the curve presents a higher probability of a treatment being the better intervention. We estimated the inconsistency of direct and indirect evidence by global inconsistency and node-splitting methods. *P* < 0.05 indicated significant heterogeneity in the entire network. The statistical analysis software was STATA 15.0 (Stata Corporation, College Station, TX, USA) with “network” packages.

### Availability of data and materials

Raw data can be accessed in the Supplementary Materials.

## RESULTS

### Characteristics of included studies

**Figure 1:**
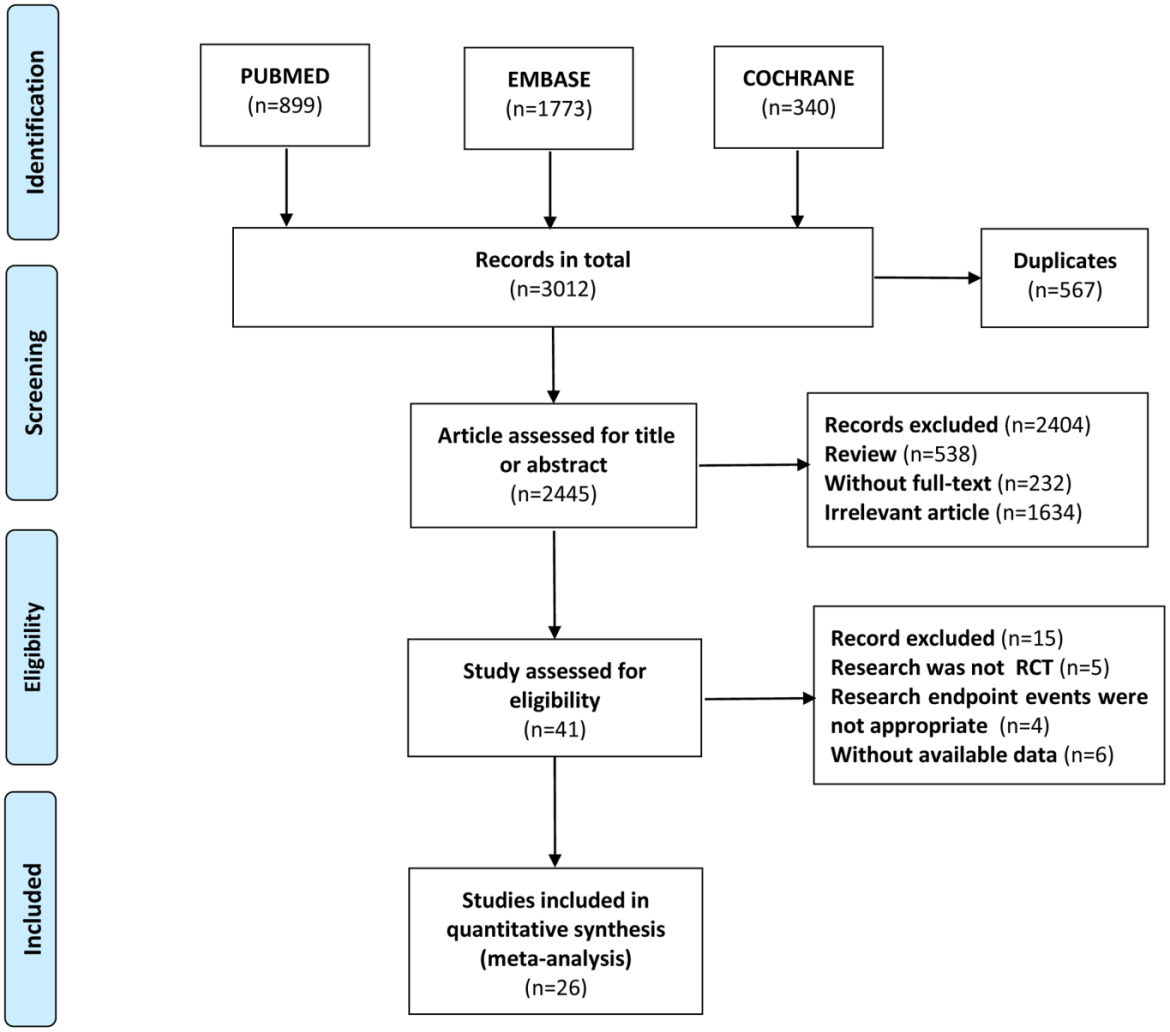
**Flowchart of the study.** The study followed the Preferred Reporting Items for Systematic Reviews and Meta-Analyses (PRISMA) guidelines.

Twenty-six RCTs involving 14945 participants met our inclusion criteria (Figure 1). Table 1 and Supplementary Table 1 show the characteristics of included studies. The trial sample size ranged from 37 to 3554, and the mean age of patients ranged from 48 to 66 years. The length of their intervention ranged from 4 to 148 weeks. 44.42% (*n* = 6639) were women. Of the 26 studies in the network analysis, 7 RCTs enrolled 998 patients undergoing peritoneal dialysis, and the remaining patients were undergoing hemodialysis. This network analysis evaluated five kinds of HIF-PHIs (roxadustat, daprodustat, vadadustat, molidustat, and enarodustat) and two kinds of ESAs (epoetin and darbepoetin). It contained the following comparisons: HIF-PHIs vs. placebo/control (*n* = 7), HIF-PHIs vs. epoetin (*n* = 9), HIF-PHIs vs. darbepoetin (*n* = 6), HIF-PHIs vs. ESAs (epoetin and darbepoetin) (*n* = 4).

**Table 1:** Characteristics of the include studies.

**Author /year**	**Size**	**Follow-up**	**Age**	**Study type**	**Interventions (no.)**	**Comparisons**	**Outcomes**	**Measures**	**Risk of bias**
Akizawa/2017 [40] NCT02019719	97	4 weeks	62y	RCT	Daprodustatt (*n* = 78) Placebo (*n* = 19)	Daprodustatt vs. Placebo	Hemoglobin level; Hypertension Iron parameters	Mean ± SD Odds ratio	low risk
Akizawa/2020 [41] NCT02969655	271	52 weeks	64y	RCT	Daprodustatt (*n* = 136) Darbepoetin Alfa (*n* = 135)	Daprodustatt vs. Darbepoetin Alfa	Hemoglobin level; Hypertension Iron parameters	Mean ± SD Odds ratio	low risk
Bailey/2019 [42] NCT02689206	97	4 weeks	64y	RCT	Daprodustatt (*n* = 79) Placebo (*n* = 18)	Daprodustatt vs. Placebo	Hemoglobin level; Hypertension Thrombosis	Mean ± SD Odds ratio	low risk
Brigandi/2016 [43] NCT01047397	37	8 weeks	60y	RCT	Daprodustatt (*n* = 31) placebo (*n* = 6)	Daprodustatt vs. Placebo	Hemoglobin level	Mean ± SD	high risk
Holdstock/2016 [44] NCT01587924	82	4 weeks	58y	RCT	Daprodustatt (*n* = 62) rhEPO (*n* = 20)	Daprodustat vs. rhEPO	Hemoglobin level; Iron parameters	Mean ± SD	low risk
Meadowcroft/2018 [45] NCT01977482	210	24 weeks	60y	RCT	Daprodustatt (*n* = 171) Control (*n* = 39)	Daprodustat vs. Control	Hemoglobin level; Hypertension Iron parameters	Mean ± SD Odds ratio	low risk
Singh/2021 [46] NCT02879305	2964	148 weeks	58y	RCT	Daprodustatt (*n* = 1487) ESA (*n* = 1477)	Daprodustat vs. ESA	Hemoglobin level; Hypertension Thrombosis; Iron parameters	Mean ± SD Odds ratio	high risk
Akizawa/2020 [47] NCT02952092	301	24 weeks	65y	RCT	Roxadustat (*n* = 150) Darbepoetin alfa (*n* = 151)	Roxadustat vs. Darbepoetin alfa	Hemoglobin level; Iron parameters	Mean ± SD	low risk
Barratt/2021 [48] NCT02278341	2796	36 weeks	56y	RCT	Roxadustat (*n* = 1379) ESA (*n* = 1417)	Roxadustat vs. ESA	Hemoglobin level	Mean ± SD	high risk
Chen/2017 [29] NCT01596855	96	8 weeks	62y	RCT	Roxadustat (*n* = 74) rhEPO (*n* = 22)	Roxadustat vs. rhEPO	Hemoglobin level; Hypertension Iron parameters	Mean ± SD Odds ratio	high risk
Chen/2019 [27] NCT02652806	304	27 weeks	49y	RCT	Roxadustat (*n* = 204) Epoetin alfa (*n* = 100)	Roxadustat vs. Epoetin alfa	Hemoglobin level; Hypertension Iron parameters	Mean ± SD Odds ratio	high risk
Chen/2021 [49]	50	36 weeks	48y	RCT	Roxadustat (*n* = 12) EPO (*n* = 38)	Roxadustat vs. EPO	Hemoglobin level; Iron parameters	Mean ± SD	high risk
Csiky/2021 [50] NCT02964936	836	52 weeks	61y	RCT	Roxadustat (*n* = 415) ESA (*n* = 421)	Roxadustat vs. ESA	Hemoglobin level; Hypertension Thrombosis; Iron parameters	Mean ± SD Odds ratio	high risk
Hou/2021 [51]	129	24 weeks	48y	RCT	Roxadustat (*n* = 86) ESAs (*n* = 43)	Roxadustat vs. ESAs	Hemoglobin level; Hypertension Iron parameters	Mean ± SD Odds ratio	low risk
Provenzano/2016 [30] NCT02273726	90	19 weeks	57y	RCT	Roxadustat (*n* = 67) Epoetin alfa (*n* = 23)	Roxadustat vs. Epoetin alfa	Hemoglobin level; Iron parameters	Mean ± SD	high risk
Provenzano/2021 [52] NCT02052310 NCT02174731	1043 2019	52 weeks 52 weeks	54y 54y	RCT RCT	Roxadustat (*n* = 522) Epoetin alfa (*n* = 521) Roxadustat (*n* = 1003) EPO (*n* = 1016)	Roxadustat vs. Epoetin alfa Roxadustat vs. EPO	Hemoglobin level; Hypertension Thrombosis; Iron parameters Hemoglobin level; Hypertension	Mean ± SD Odds ratio Mean ± SD Odds ratio	high risk
Akizawa/2019 [53]	74	17 weeks	60y	RCT	Molidustat (*n* = 59) Control (*n* = 15)	Molidustat vs. Control	Hemoglobin level	Mean ± SD	high risk
Akizawa/2019 [54] NCT02064426	87	52 weeks	60y	RCT	Molidustat (*n* = 57) Epoetin (*n* = 30)	Molidustat vs. Epoetin	Hemoglobin level; Hypertension Thrombosis; Iron parameters	Mean ± SD Odds ratio	high risk
Akizawa/2021 [55] NCT03543657	229	52 weeks	66y	RCT	Molidustat (*n* = 153) Darbepoetin (*n* = 76)	Molidustat vs. Darbepoetin	Hemoglobin level; Hypertension Iron parameters	Mean ± SD Odds ratio	low risk
Macdougall/2018 [56] NCT01975818b	199	16 weeks	59y	RCT	Molidustat (*n* = 157) Epoetin (*n* = 42)	Molidustat vs. Epoetin	Hemoglobin level; Hypertension Iron parameters	Mean ± SD Odds ratio	low risk
Akizawa/2019 [57]	82	24 weeks	62y	RCT	Enarodustat (*n* = 60) Placebo (*n* = 22)	Enarodusta vs. Placebo	Hemoglobin level; Iron parameters	Mean ± SD	low risk
Akizawa/2021 [58]	172	24 weeks	64y	RCT	Enarodustat (*n* = 86) Darbepoetin alfa (*n* = 86)	Enarodusta vs. Darbepoetin alfa	Hemoglobin level; Hypertension Thrombosis; Iron parameters	Mean ± SD Odds ratio	low risk
Eckardt/2021 [59] NCT02892149	3554	116 weeks	58y	RCT	Vadadustat (*n* = 1777) Darbepoetin alfa (*n* = 1777)	Vadadustat vs. Darbepoetin alfa	Hemoglobin level; Hypertension Thrombosis	Mean ± SD Odds ratio	high risk
Nangaku/2020 [60] NCT03054350	58	18 weeks	64y	RCT	Vadadustat (*n* = 44) Placebo (*n* = 14)	Vadadusta vs. Placebo	Hemoglobin level; Iron parameters	Mean ± SD	low risk
Nangaku/2021 [61] NCT03439137	323	52 weeks	65y	RCT	Vadadustat (*n* = 162) Darbepoetin alfa (*n* = 161)	Vadadustat vs. Darbepoetin alfa	Hemoglobin level; Hypertension Thrombosis; Iron parameters	Mean ± SD Odds ratio	low risk

Abbreviations: RCT: randomized controlled trial; SD: standard deviation; ESA: erythropoiesis-stimulating agent; EPO: erythropoietin.

### Risk of bias

According to the Cochrane Collaboration tool, most studies showed a low risk of bias. Twelve studies reported a high risk of bias because they were not double-blinded (Supplementary Figure 1).

### Network meta-analysis

**Figure 2:**
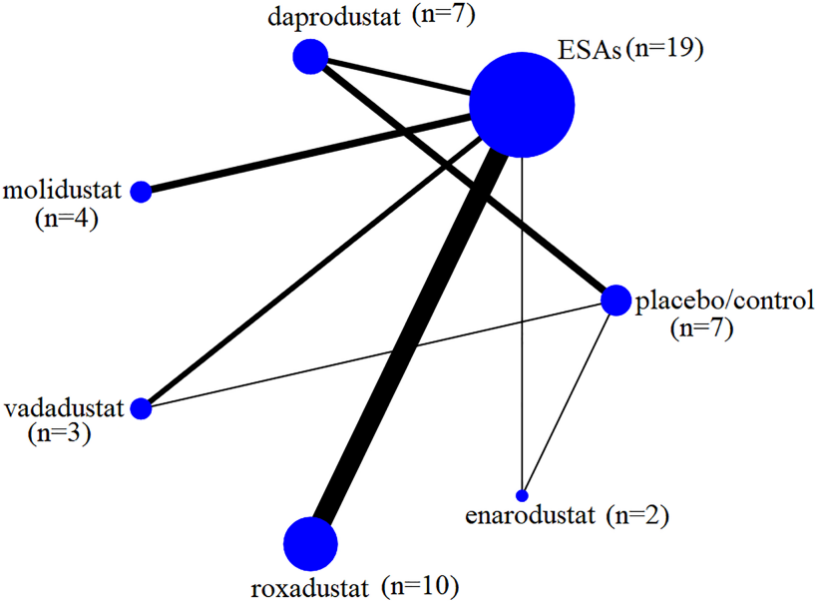
**Network of the study treatments.** Nodes represent intervention comparisons. The size of the nodes is proportional to participant numbers. The width of the lines was in direct ratio to the number of trials.

Figure 2 shows the network of all comparisons. These trials included epoetin (*n* = 9), darbepoetin (*n* = 6), ESAs (*n* = 4), daprodustat (*n* = 7), molidustat (*n* = 4), vadadustat (*n* = 3), roxadustat (*n* = 10), enarodustat (*n* = 2), and placebo/control (*n* = 7).

### Inconsistency analysis

We used a node-splitting approach and a global inconsistency analysis to examine the consistency of all comparisons. The global inconsistency result shows no statistical inconsistency (*P* = 0.377). The outcomes of the node-splitting method are shown in Supplementary Table 2. Both direct and indirect comparisons were consistent.

### Changes in hemoglobin levels from baseline

**Figure 3:**
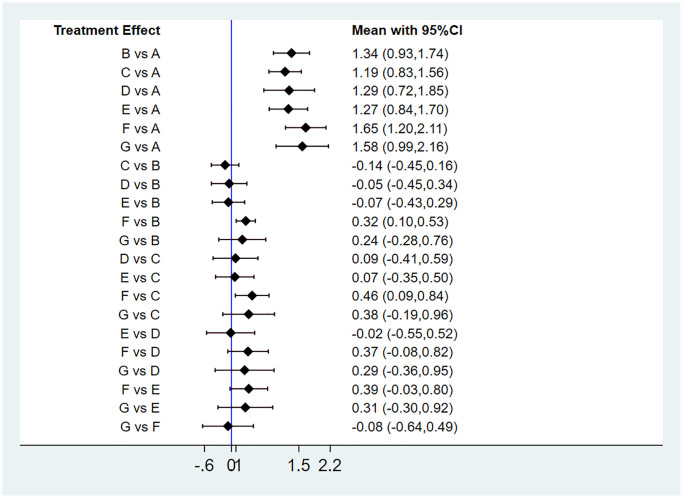
**Forest plot of hemoglobin levels.** Abbreviations: A: placebo/control; B: ESAs; C: daprodustat; D: molidustat; E: vadadustat; F: roxadustat; G: enarodustat.

Twenty-six RCTs reported data on hemoglobin. Figure 3 shows the results of the network meta-analysis for hemoglobin. Compared with a placebo, five HIF-PHIs and ESAs significantly increased hemoglobin. Roxadustat significantly increased hemoglobin more than ESAs (0.32, 0.10 to 0.53) and daprodustat (0.46, 0.09 to 0.84). The other four HIF-PHIs did not improve hemoglobin levels more effectively than ESAs. There was also no statistical difference among daprodustat, molidustat, vadadustat, and enarodustat.

**Figure 4:**
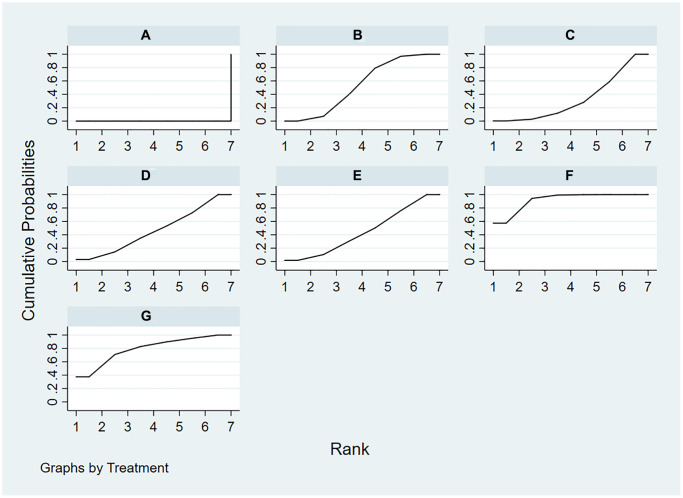
**The surface under the cumulative ranking curve for hemoglobin.** (**A**) placebo/control; (**B**) ESAs; (**C**) daprodustat; (**D**) molidustat; (**E**) vadadustat; (**F**) roxadustat; (**G**) enarodustat.

Supplementary Table 3 presents ranking probabilities, ranking plots, mean ranks, and SUCRA values for all interventions. In hemoglobin levels, roxadustat was ranked as the best treatment with a SUCRA value of 91.8%. The second-best treatment was enarodustat (79.4%), and the third-best was ESAs (53.9%). The intervention with the lowest SUCRA value was daprodustat (33.7%) (Figure 4).

### Changes in iron parameters from baseline

We analyzed the influence of HIF-PHIs and ESAs on iron parameters, including ferritin, hepcidin, TIBC, TSAT, and serum iron. In all intervention comparisons, serum iron changes weren’t statistically significant. Compared with the placebo, ESAs and five HIF-PHIs significantly reduced TSAT. For other iron parameters, ESAs and molidustat didn’t affect. Vadadustat and enarodustat elevated TIBC (SMD 3.90, 95% CI 2.24 to 5.56; 2.35, 0.74 to 3.97) and decreased hepcidin (MD −38.79, 95% CI −68.95 to −8.63; −54.95, −91.21 to −18.69). Daprodustat increased TIBC (SMD 1.24, 95% CI 0.17 to 2.31) and reduced ferritin (MD −97.72, 95% CI −187.72 to −7.71) as well as hepcidin (MD −46.02, 95% CI −75.64 to −16.41). Roxadustat reduced ferritin (MD −145.68, 95% CI −256.90 to −34.47) and hepcidin (MD −47.73, 95% CI −82.78 to −12.68). In conclusion, TSAT concentrations were reduced by HIF-PHIs and ESAs. Roxadustat and daprodustat decreased ferritin. Four drugs reduced hepcidin: daprodustat, vadadustat, roxadustat, and enarodustat. Vadadustat, daprodustat, and enarodustat all had significantly higher TIBC levels. Comparing these four HIF-PHIs, ferritin, hepcidin, TSAT, and serum iron levels weren’t significantly changed. Vadadustat increased TIBC much more than daprodustat (SMD 2.66, 95% CI 0.94 to 4.38) (Supplementary Figures 2–6).

SUCRA ranked all groups in the influence on the iron parameters (Supplementary Figures 7–11 and Supplementary Table 3). Roxadustat was ranked first in decreasing ferritin and second in reducing hepcidin, with SUCRA values of 90.9% and 74%. Enarodustat was ranked first in the reduction of hepcidin (80.9%) and TSAT (88.7%). Vadadustat performed the best effect on increased TIBC (98.7%).

### Hypertension and thrombosis

A lower incidence of hypertension was observed in vadadustat than in roxadustat (1.36, 1.01 to 1.82) and ESAs (0.74, 0.60 to 0.91). Daprodustat (2.52, 1.28 to 4.94) and roxadustat (1.61, 1.22 to 2.12) induced higher thrombosis than ESAs (Supplementary Figures 12 and 13). From the SUCRA value, we found that the top two drugs with the lowest risk of hypertension were molidustat (80.7%) and vadadustat (76.5%). ESAs (88.5%) and enarodustat (67.9%) ranked first two positions, respectively, in the safety of thrombosis (Supplementary Figures 14 and 15).

### Small-study effect analysis

We found no evidence of small study effects across outcomes (Figure 5, Supplementary Figures 16–22).

**Figure 5:**
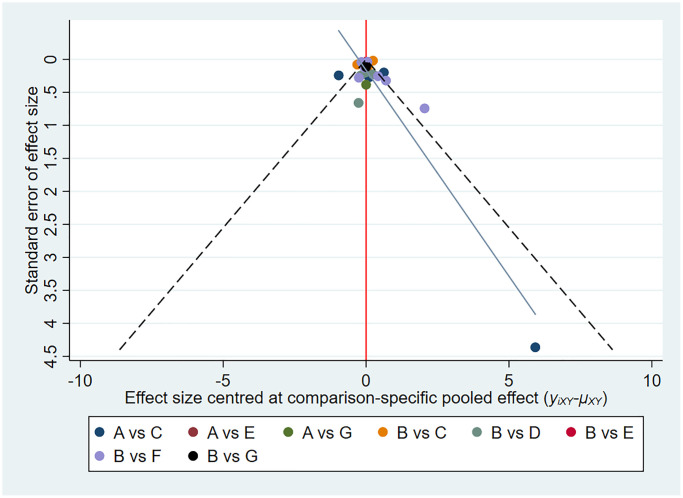
**Funnel plots assessing hemoglobin levels.** The red line represents the null hypothesis. The yellow/blue line represents the regression line. Different colours correspond to different comparisons. Abbreviations: A: placebo/control; B: ESAs; C: daprodustat; D: molidustat; E: vadadustat; F: roxadustat; G: enarodustat.

## DISCUSSION

### Principal findings

This systematic review and network meta-analysis demonstrated the impact of ESAs and HIF-PHIs on clinical outcomes in patients with CKD-DD. Roxadustat performed significantly more than ESAs and daprodustat and ranked first in hemoglobin improvement. According to the SUCRA ranking, roxadustat and daprodustat were the most effective medications for reducing ferritin. For lowering hepcidin levels, enarodustat and roxadustat came in first and second, respectively. The previous two medications for the improvement of TIBC were vadadustat and enarodustat. Enarodustat and ESAs had the lowest TSAT of all the drugs. Daprodustat and roxadustat lead to higher thrombosis events than ESAs. Molidustat and ESAs were the safest drugs with the lowest hypertension and thrombosis risk.

### Comparison with other studies

This study provided sufficient evidence supporting the efficacy of five HIF-PHIs in hemoglobin improvement in CKD-DD patients. Roxadustat performed better efficacy in elevating hemoglobin levels than ESAs in CKD-DD patients. This result is consistent with previous meta-analyses [23–25]. One meta-analysis investigated roxadustat on hemoglobin in six studies [26–31]. It presented that in CKD-DD patients, hemoglobin levels increased in roxadustat (0.52, 0.38 to 0.66) compared to EPO. The other meta-analysis included ten RCTs with 3031 patients in the roxadustat group and 2737 in the ESAs group. It demonstrated that roxadustat was associated with increased hemoglobin levels (0.2, 0.02 to 0.39, *P* = 0.03) compared with the ESAs group. A meta-analysis investigated daprodustat in CKD-DD patients [32]. It reported that hemoglobin change was significantly higher in the daprodustat group than that in the placebo (1.88, 0.68 to 3.09, *P* = 0.002). There was no significant difference between daprodustat and EPO (0.12, −0.28 to 0.52, *P* = 0.55). This study supports this finding. Moreover, this study discovered that roxadustat is much more effective in hemoglobin improvement than daprodustat in CKD-DD patients. Although roxadustat ranked the best medical intervention in hemoglobin, its higher risk of hypertension and thrombosis can’t be ignored [33, 34]. Higher hemoglobin level is a risk factor for thrombosis. The increase in hemoglobin will raise blood viscosity, slow blood flow, and possibly result in thrombosis. High hemoglobin may be hyperplastic cell disease, which may cause platelet rise and thrombosis. The APSN reported an association between iron deficiency and thrombosis events. Iron supplements probably reduce the roxadustat-related thrombosis risk. Molidustat and ESAs are appropriate drugs for patients with hypertension and thrombosis risk.

There is no meta-analysis about the efficacy and risk of vadadustat, molidustat, and enarodustat in patients with CKD-DD. This study first demonstrated the benefit of vadadustat, molidustat, and enarodustat on clinical outcomes. However, these three HIF-PHIs presented comparable hemoglobin improvement to ESAs. The top two drugs with the lowest rate of hypertension were molidustat and vadadustat, and thrombosis was ESAs and enarodustat. These results provide clinicians with evidence to select appropriate medicine considering the benefits and risks for CKD-DD patients.

Previous studies did not examine the effect of five HIF-PHIs on iron parameters in CKD-DD patients. This research investigated the impact of five HIF-PHIs on iron parameters. As an iron-regulating protein, hepcidin is essential in iron balance. Ferroportin is the only known exporter of iron from mammalian cells. Hepcidin degrades ferroportin to inhibit the release of stored iron from reticuloendothelial cells. Therefore, hepcidin-mediated iron depletion limits erythropoiesis [35–37]. Patients with CKD-DD experience an increase in hepcidin, particularly during inflammation. Higher hepcidin levels correlate with lower hemoglobin levels and a greater likelihood of anemia [38, 39]. Except for molidustat, HIF-PHIs in this study reduce hepcidin. ESAs do not participate in the regulation of hepcidin. Therefore, this study suggested that HIF-PHIs, except for molidustat, could replace ESAs in treating CKD-DD patients with inflammation or high hepcidin levels. Enarodustat and roxadustat were the two HIF-PHIs most effective at lowering hepcidin.

Daprodustat and roxadustat both showed reduced ferritin levels. As a form of iron storage in the liver, ferritin monitoring is important in daprodustat and roxadustat administration. Clinicians can evaluate the necessity of the iron supplement by ferritin examination. Besides ferritin, TSAT and serum iron are the most common biomarkers of stored iron. In patients with functional iron deficiency on ESA therapy, enteral iron absorption or release from reticuloendothelial cells is insufficient to meet erythropoiesis demands. These patients frequently exhibit low TSAT results and may benefit from IV iron treatment. In all HIF-PHIs and ESAs, the effectiveness of lowering TSAT was observed in this study. We should focus on the TSAT levels to determine whether patients with functional iron deficiency need IV iron supplements. Consistent with a report [16], HIF-PHIs and ESAs showed no significant change in serum iron. This evidence indicates that only serum iron measure is insufficient to evaluate patients’ iron condition.

A previous network meta-analysis reported no difference in hemoglobin improvement between roxadustat and ESAs in CKD-NDD patients [22]. It is contrary to this study. This inconsistency may indicate higher hemoglobin improved by roxadustat in patients with CKD-DD than in CKD-NDD. This hypothesis achieves support from the other analysis [33]. It conducted a subgroup analysis on hemoglobin levels to compare CKD-DD patients with NDD patients. The result presented that HIF-PHIs induced higher hemoglobin than ESAs in CKD-DD patients (0.16, 0.05 to 0.27). However, HIF-PHIs weren’t significantly different from ESAs in CKD-NDD patients (−0.02, −0.26 to 0.22). This distinction between the two kinds of populations probably results from disease conditions and excretory capacity. The kidney eliminates approximately 40% of roxadustat, and roxadustat cannot be removed by dialysis. The majority of CKD-DD patients have little to no kidney function left. As a result, the roxadustat excretion rate in CKD-DD patients may be much slower than in CKD-NDD patients. However, ESA pharmacokinetics in CKD-DD patients are comparable to those in CKD-NDD patients. In CKD-DD patients, higher roxadustat concentrations probably result in better efficacy.

## CONCLUSIONS

We anticipate this study’s findings to provide implications for drug selection in renal anemia of CKD-DD patients. In treating patients with ESAs-resistant, clinicians can prescribe roxadustat to improve hemoglobin. CKD patients with inflammation and high hepcidin levels can benefit from enarodustat and roxadustat. Clinicians need to be aware of thrombosis and hypertension when prescribing roxadustat. Molidustat is a relatively safe medication for patients with hypertension. Regarding lowering thrombosis risk, ESAs are better than other therapies. Ferritin and TSAT are biomarkers for iron storage. Ferritin levels should be monitored while taking roxadustat and daprodustat. Reduced TSAT indicates that IV iron therapy is required for patients with HIF-PHIs and ESA administration.

### Limitations of this study

This study exits some limitations. First, the results of this study come from CKD-DD participants. It is not suitable for CKD-NDD patients’ applications. Second, whether iron supplements should be prescribed in CKD-DD patients during HIF-PHIs intervention is unknown.

## Supplementary Materials

Supplementary Figures

Supplementary Table 1

Supplementary Tables 2 and 3
