# Supplementary material for: A network meta-analysis of the efficacy of hypoxia-inducible factor prolyl-hydroxylase inhibitors in dialysis chronic kidney disease
Source: Aging (Albany NY). 2023 Mar 27;15(6):2237–74. doi: 10.18632/aging.204611 (PMC10085583; doi:10.18632/aging.204611)
Supplement: Supplementary Figures [file aging-15-204611-s001.pdf]

SUPPLEMENTARY FIGURES

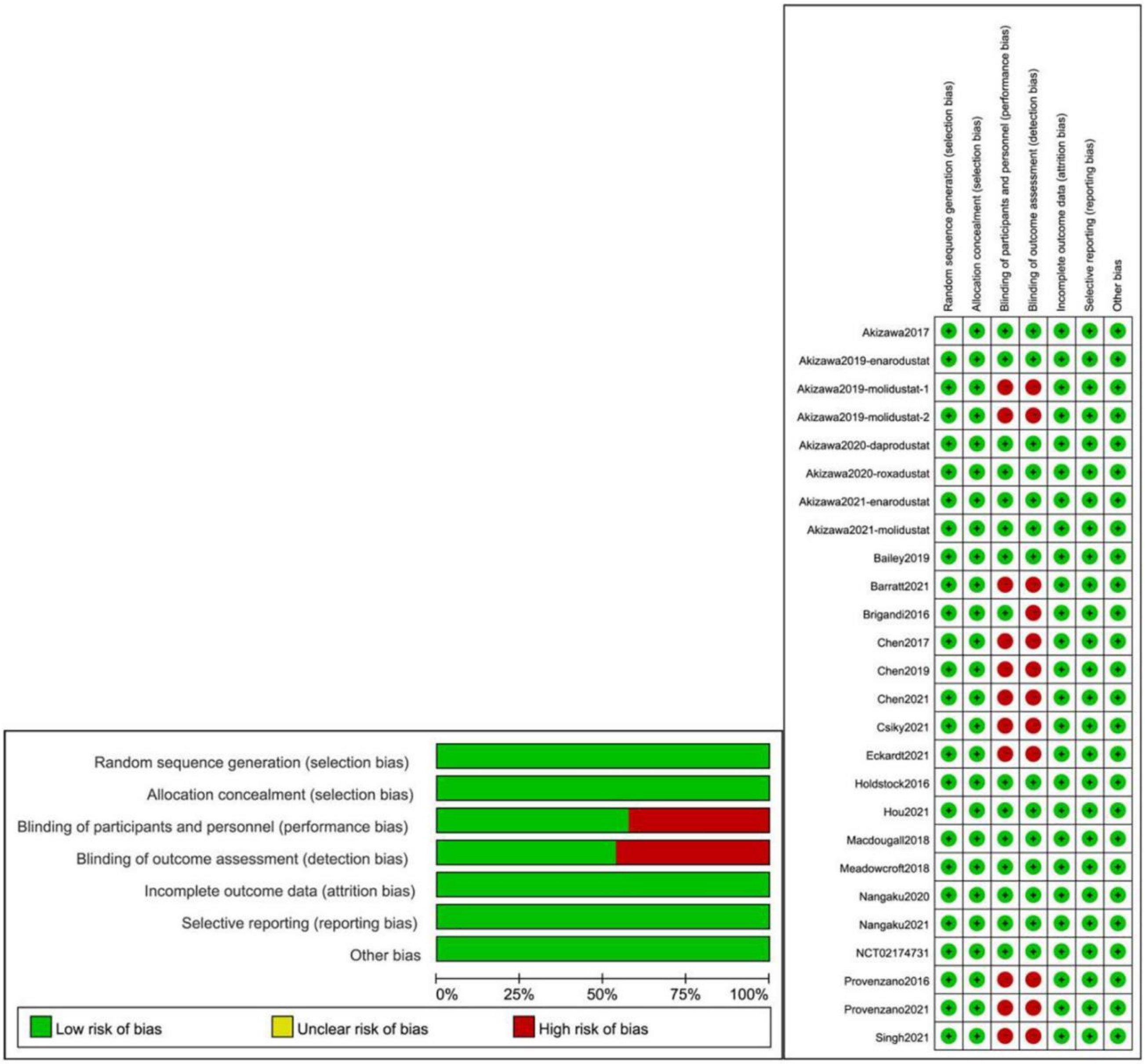

Supplementary Figure 1. Bias assessment. The Cochrane risk-of-bias tool assessed the risk of bias in included studies.

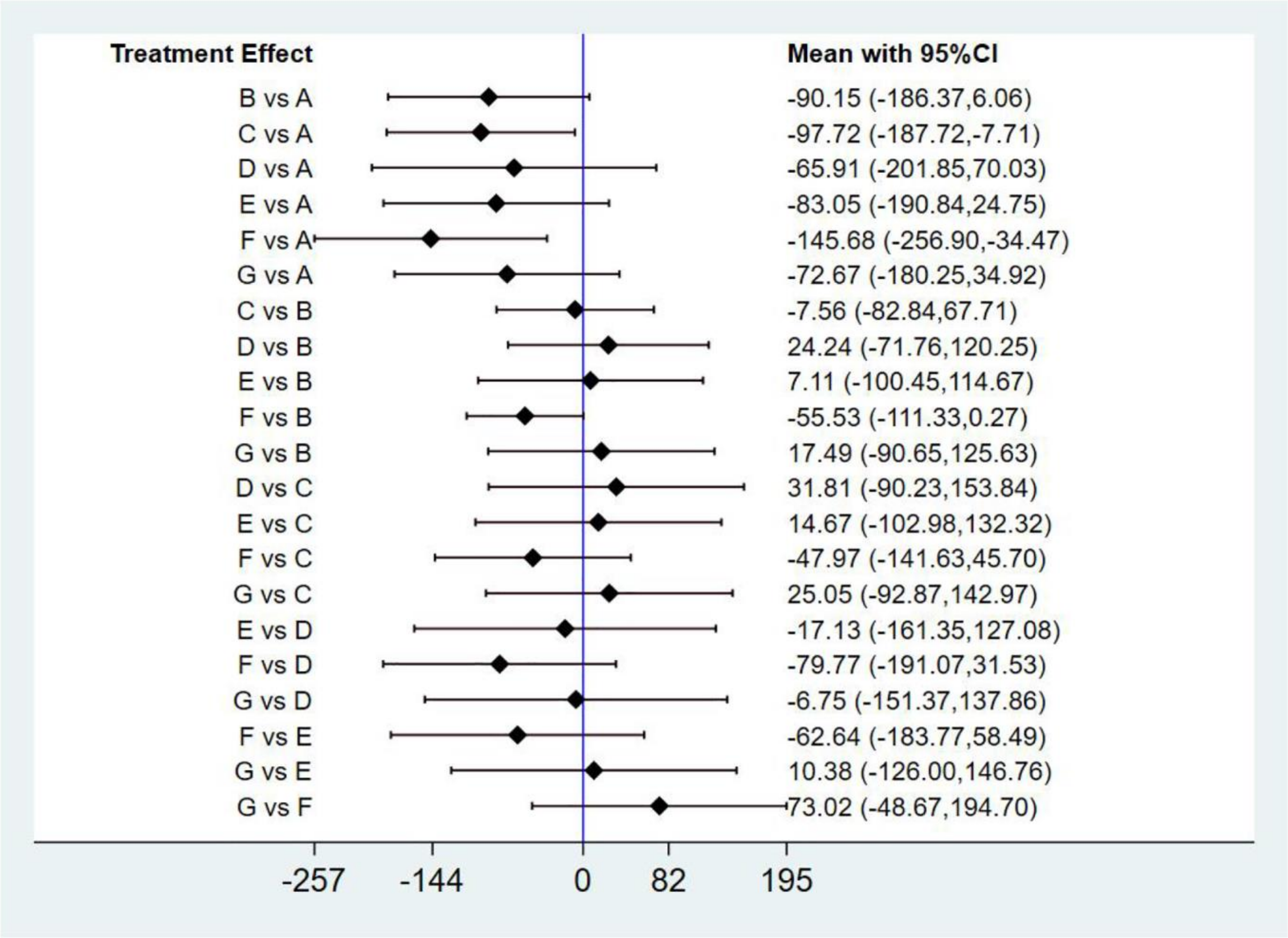

Supplementary Figure 2. Forest plot of ferritin. Abbreviations: A: placebo/control; B: ESAs; C: daprodustat; D: molidustat; E: vadadustat; F: roxadustat; G: enarodustat.

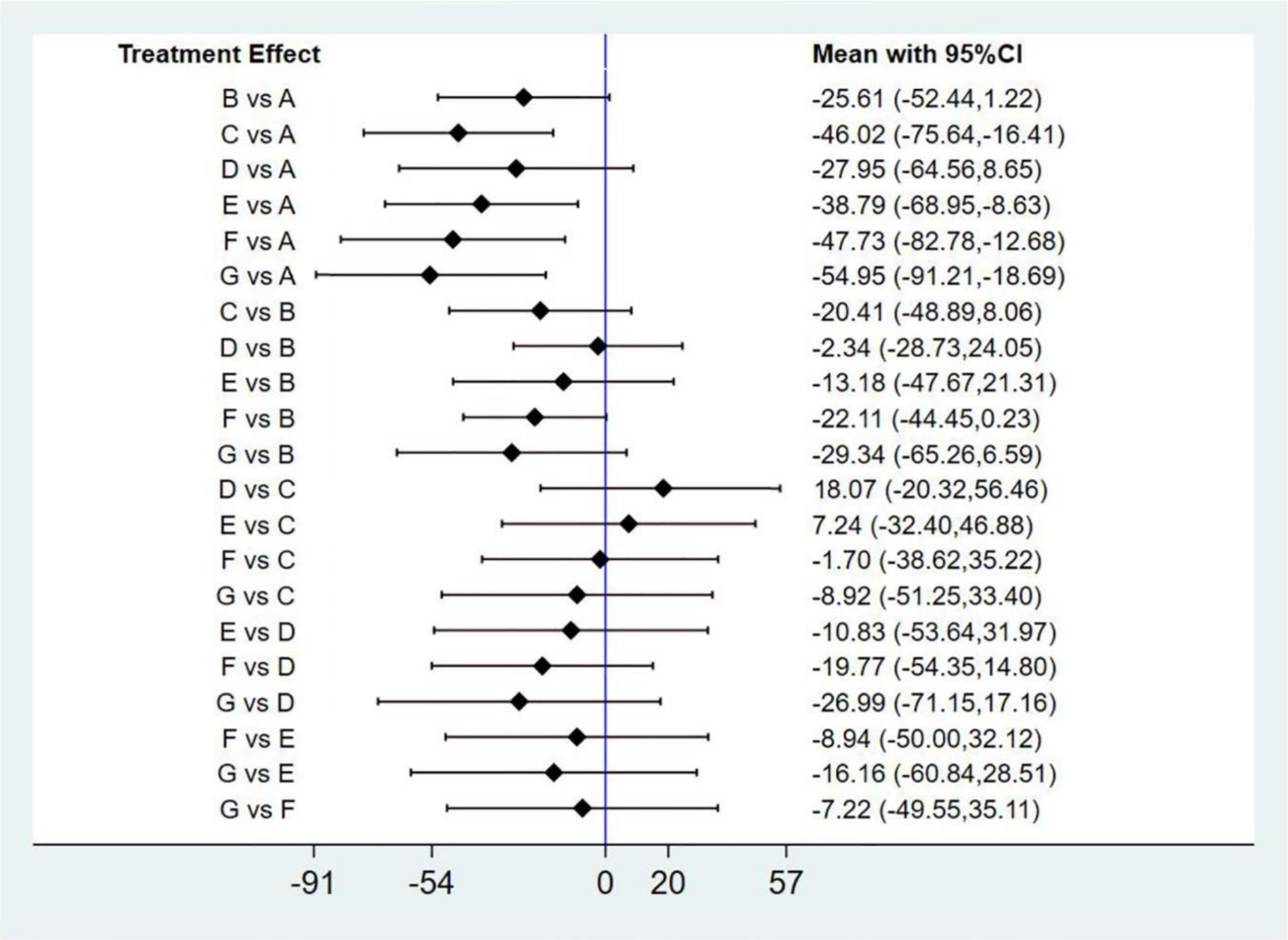

Supplementary Figure 3. Forest plots of hepcidin. Abbreviations: A: placebo/control; B: ESAs; C: daprodustat; D: molidustat; E: vadadustat; F: roxadustat; G: enarodustat.

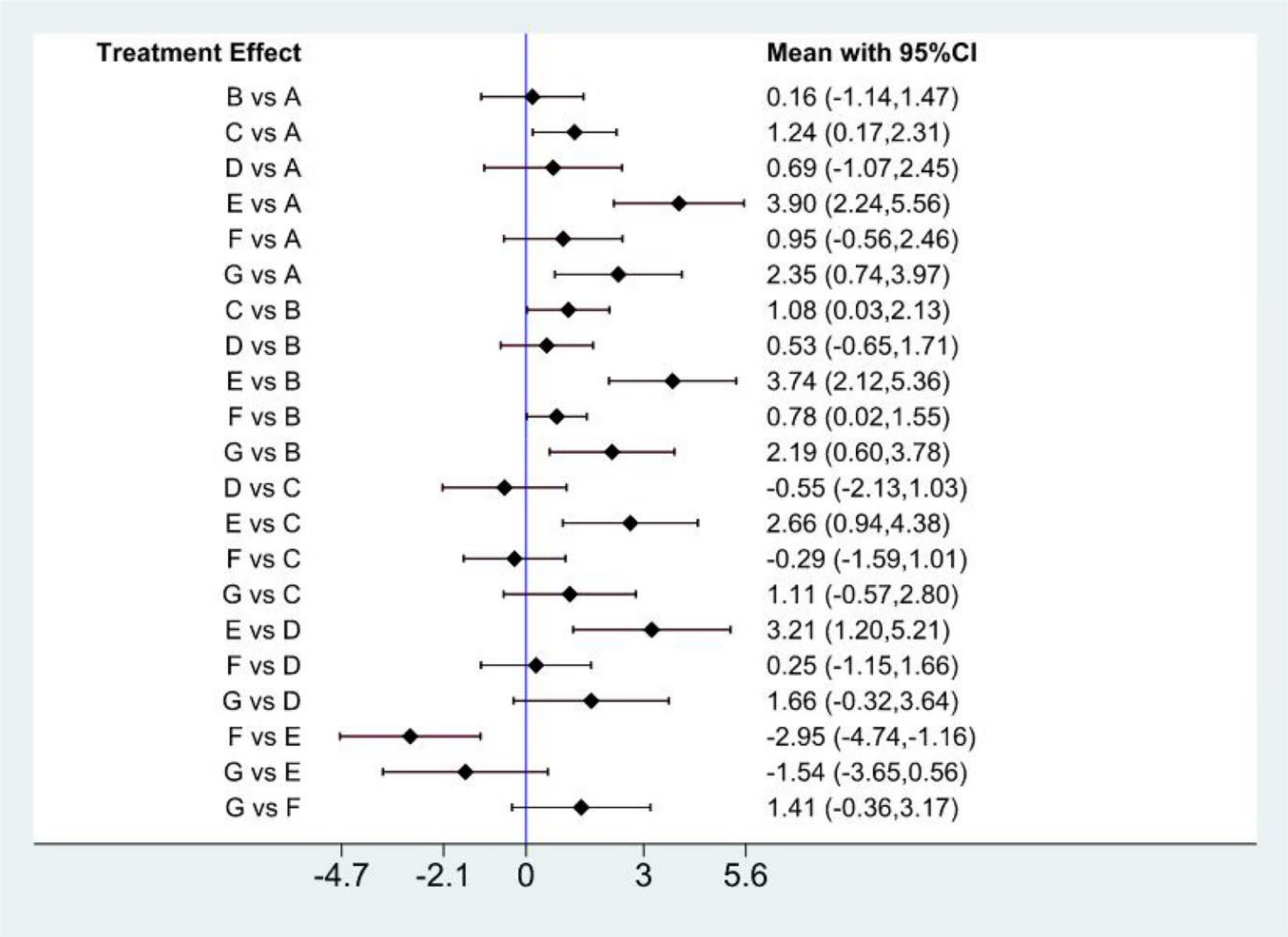

Supplementary Figure 4. Forest plots of TIBC. Abbreviations: A: placebo/control; B: ESAs; C: daprodustat; D: molidustat; E: vadadustat; F: roxadustat; G: enarodustat.

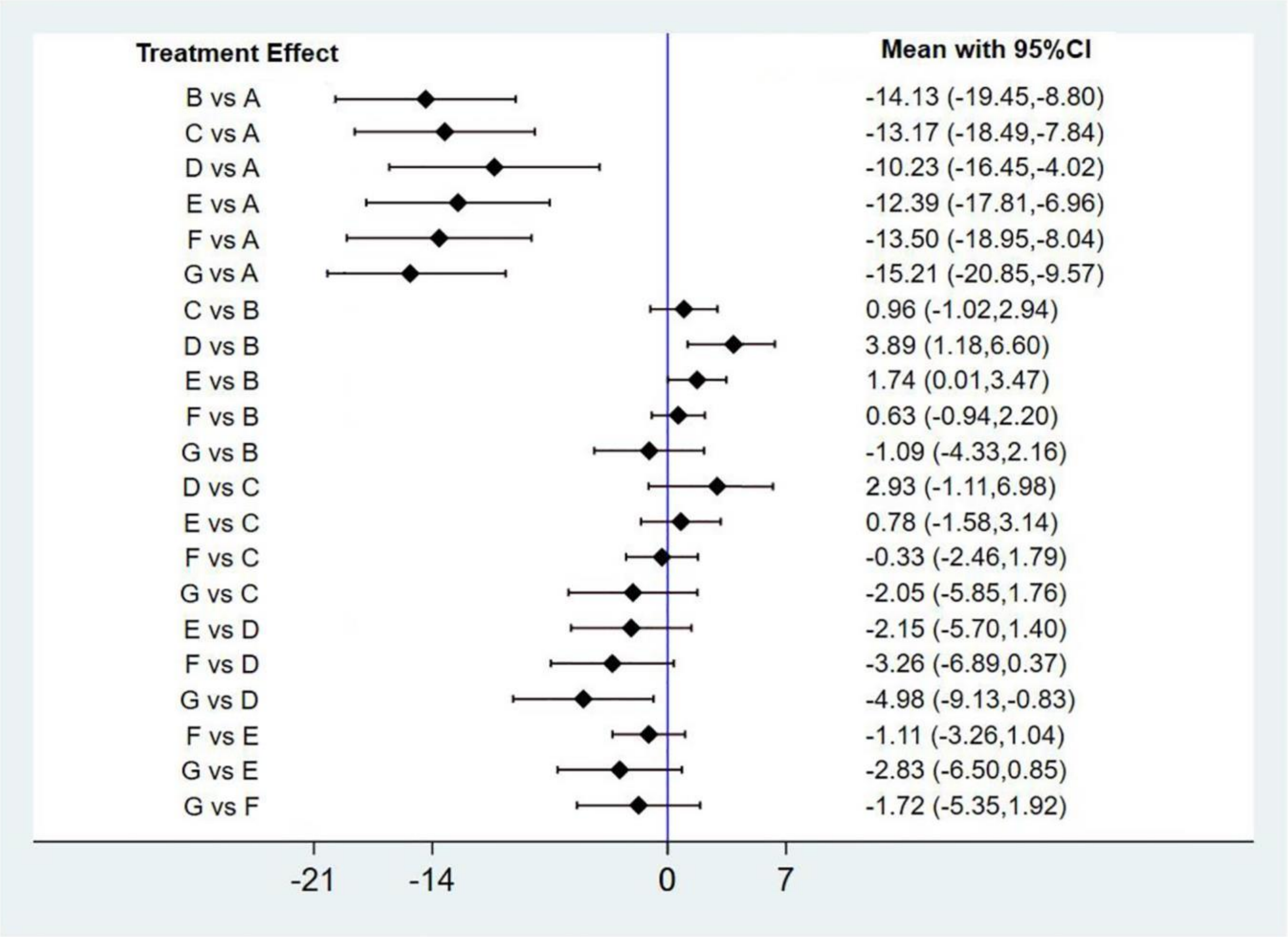

Supplementary Figure 5. Forest plots of TSAT. Abbreviations: A: placebo/control; B: ESAs; C: daprodustat; D: molidustat; E: vadadustat; F: roxadustat; G: enarodustat.

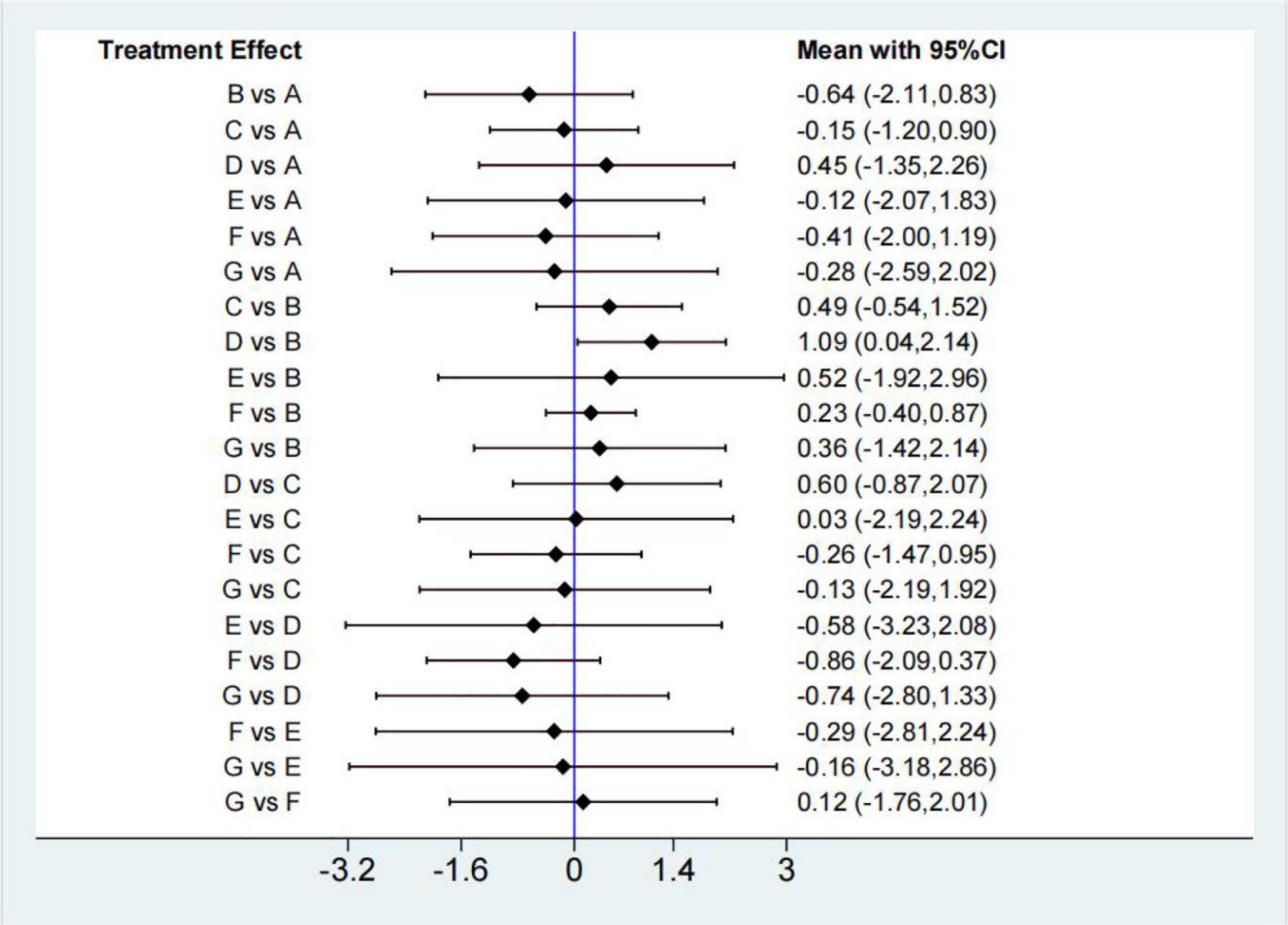

Supplementary Figure 6. Forest plots of serum iron. Abbreviations: A: placebo/control; B: ESAs; C: daprodustat; D: molidustat; E: vadadustat; F: roxadustat; G: enarodustat.

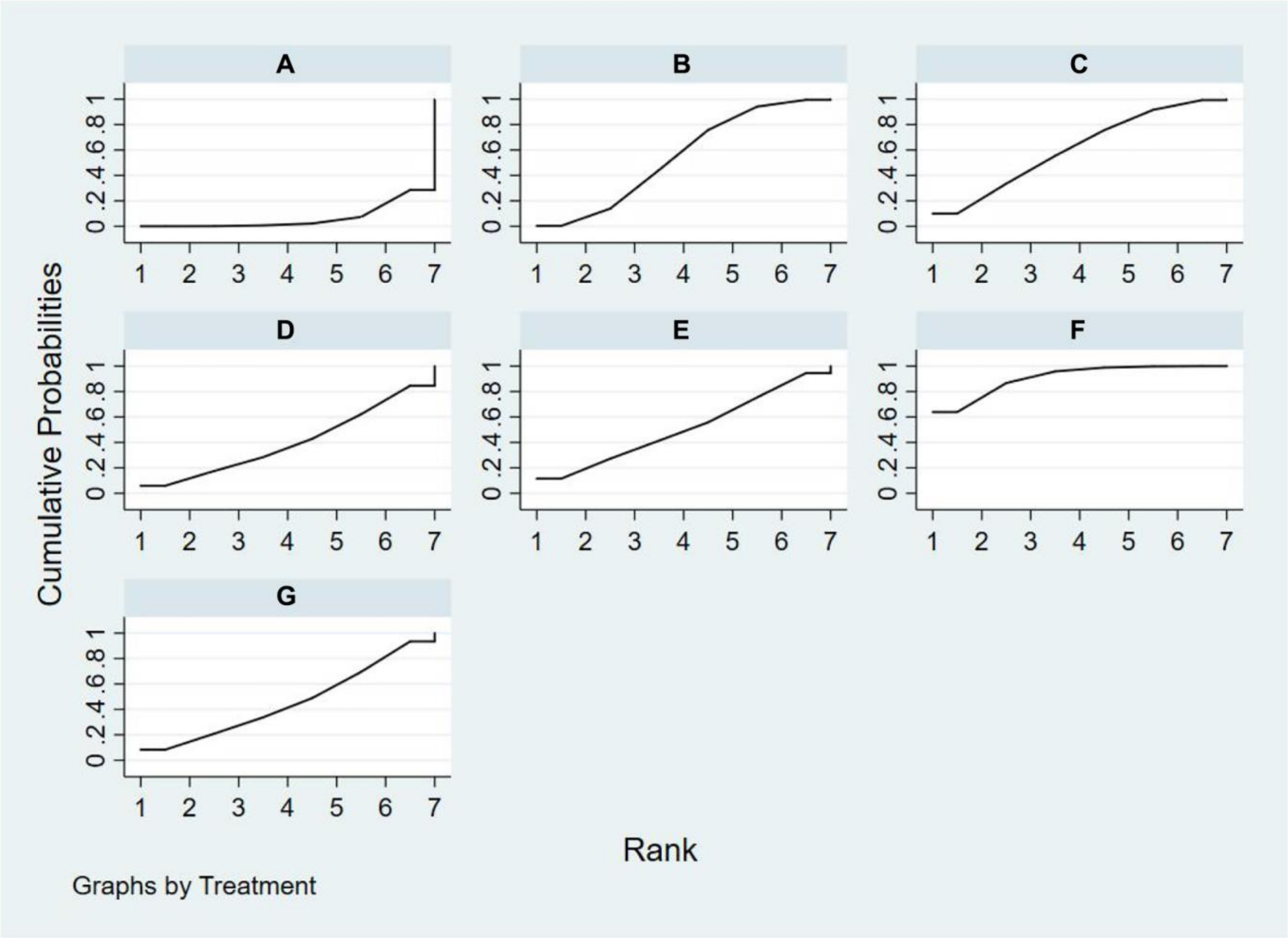

Supplementary Figure 7. The surface under the cumulative ranking curve for ferritin. (**A**) Placebo/control; (**B**) ESAs; (**C**) daprodustat; (**D**) molidustat; (**E**) vadadustat; (**F**) roxadustat; (**G**) enarodustat.

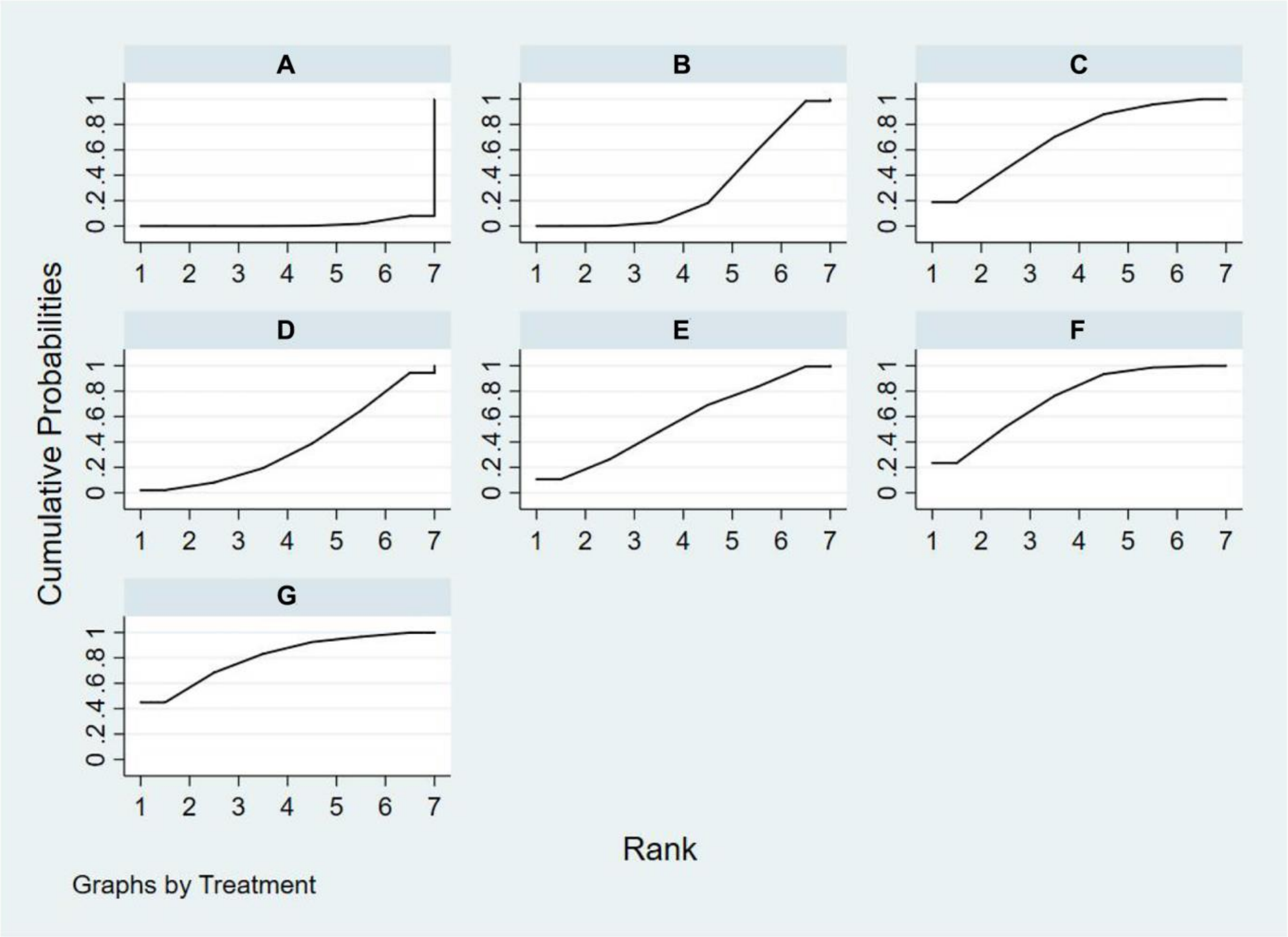

Supplementary Figure 8. The surface under the cumulative ranking curve for hepcidin. (**A**) Placebo/control; (**B**) ESAs; (**C**) daprodustat; (**D**) molidustat; (**E**) vadadustat; (**F**) roxadustat; (**G**) enarodustat.

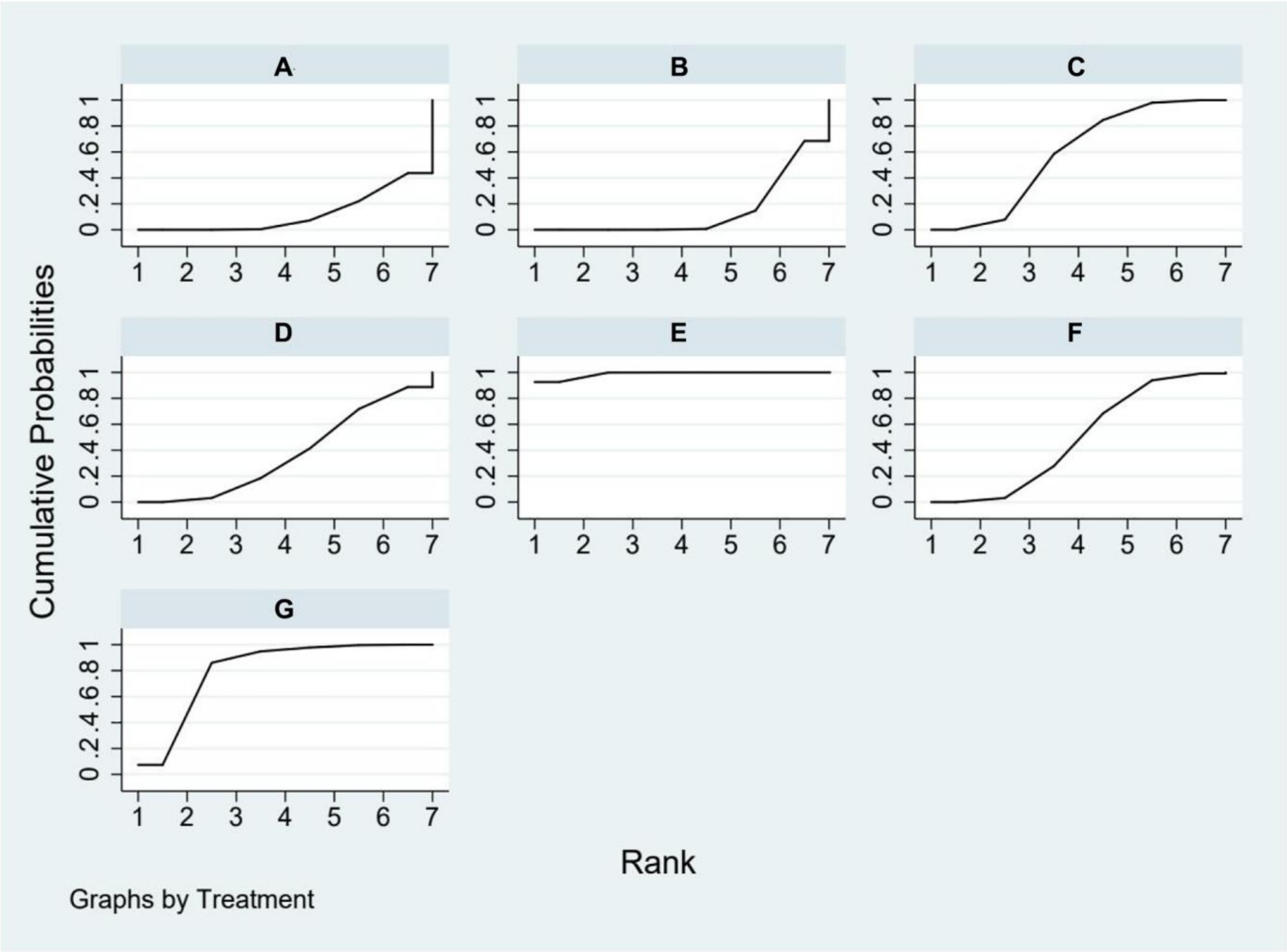

Supplementary Figure 9. The surface under the cumulative ranking curve for TIBC. (**A**) Placebo/control; (**B**) ESAs; (**C**) daprodustat; (**D**) molidustat; (**E**) vadadustat; (**F**) roxadustat; (**G**) enarodustat.

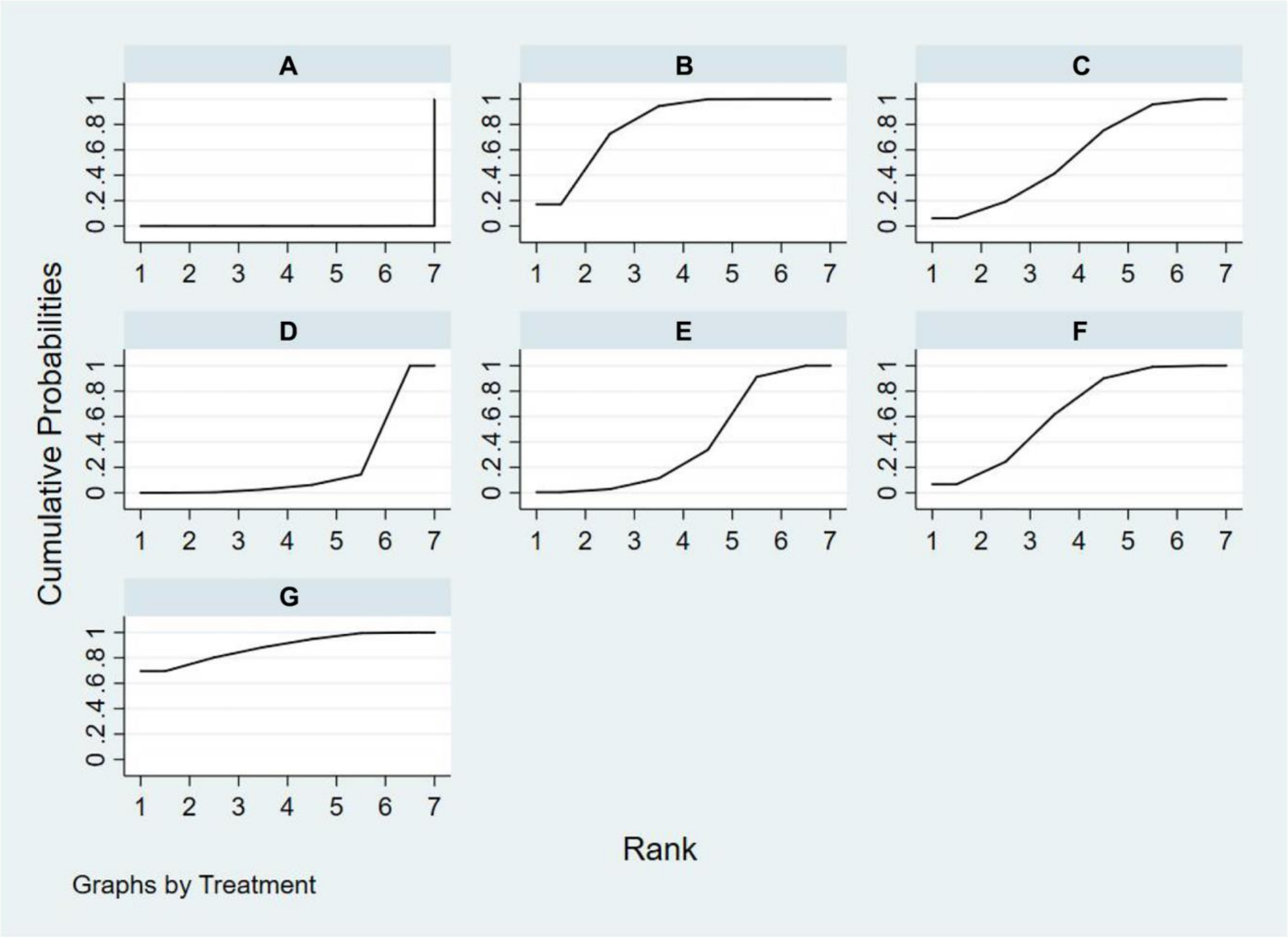

Supplementary Figure 10. The surface under the cumulative ranking curve for TSAT. (**A**) Placebo/control; (**B**) ESAs; (**C**) daprodustat; (**D**) molidustat; (**E**) vadadustat; (**F**) roxadustat; (**G**) enarodustat.

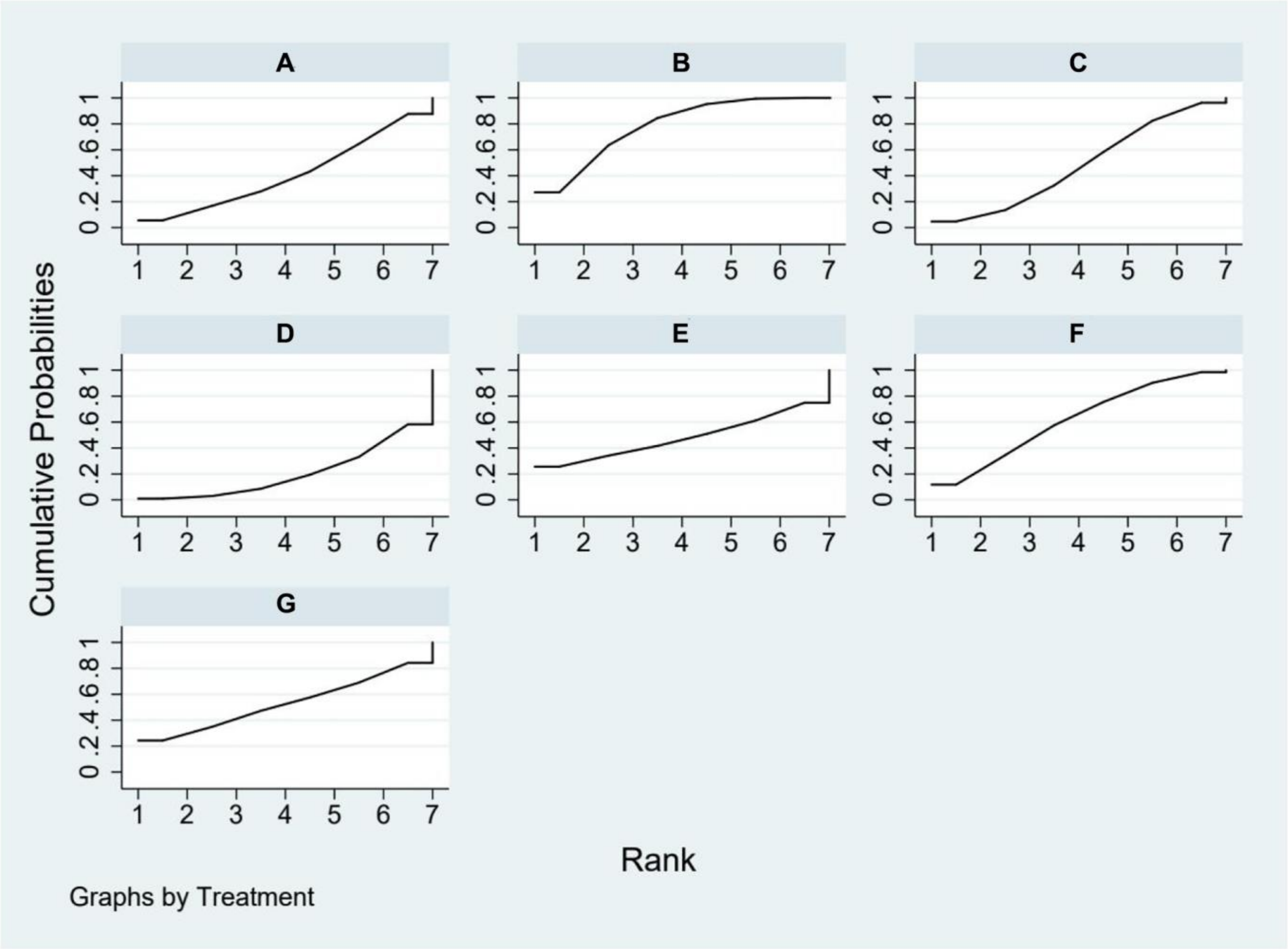

Supplementary Figure 11. The surface under the cumulative ranking curve for serum iron. (**A**) Placebo/control; (**B**) ESAs; (**C**) daprodustat; (**D**) molidustat; (**E**) vadadustat; (**F**) roxadustat; (**G**) enarodustat.

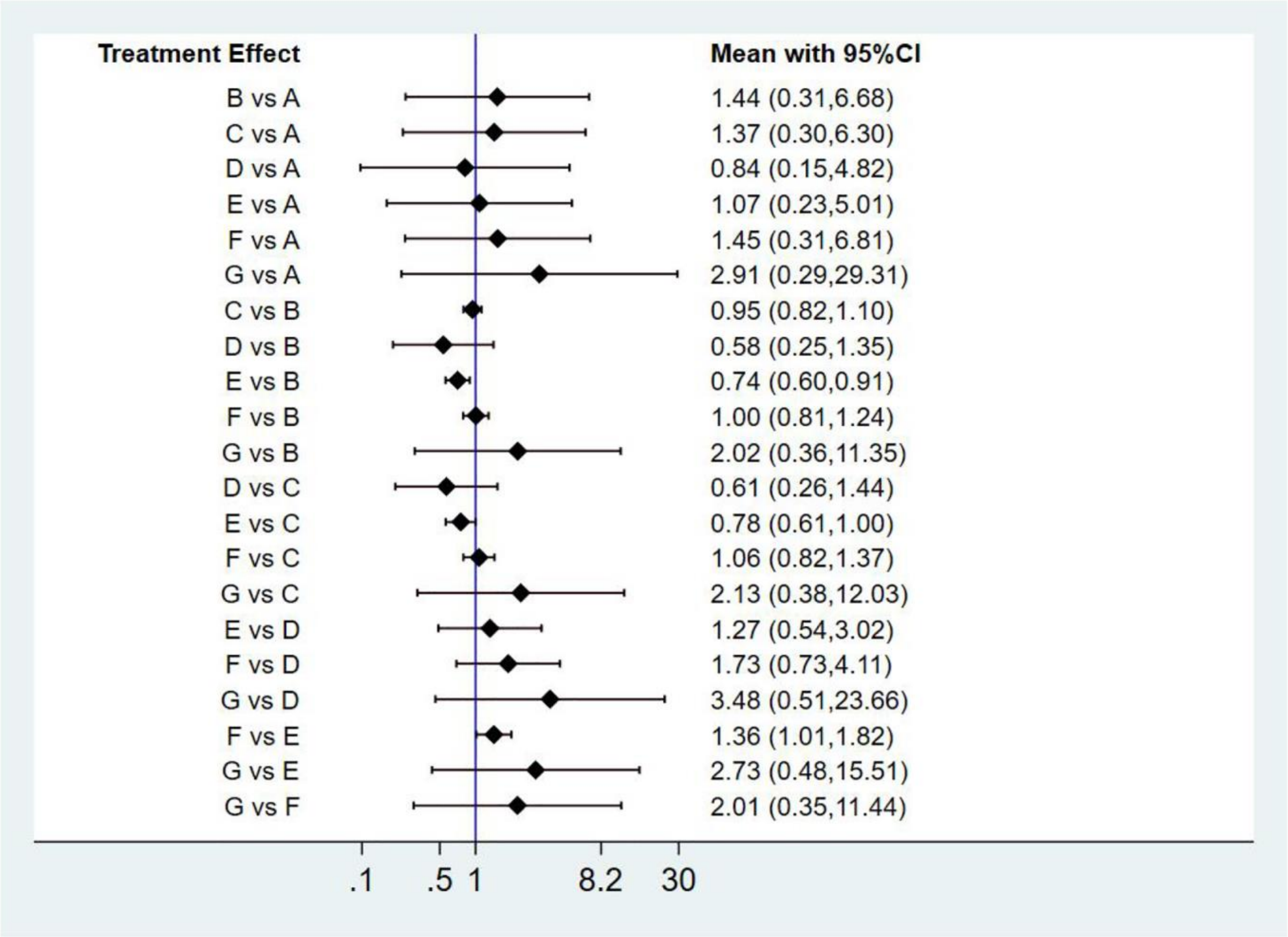

Supplementary Figure 12. Forest plots of hypertension. Abbreviations: A: placebo/control; B: ESAs; C: daprodustat; D: molidustat; E: vadadustat; F: roxadustat; G: enarodustat.

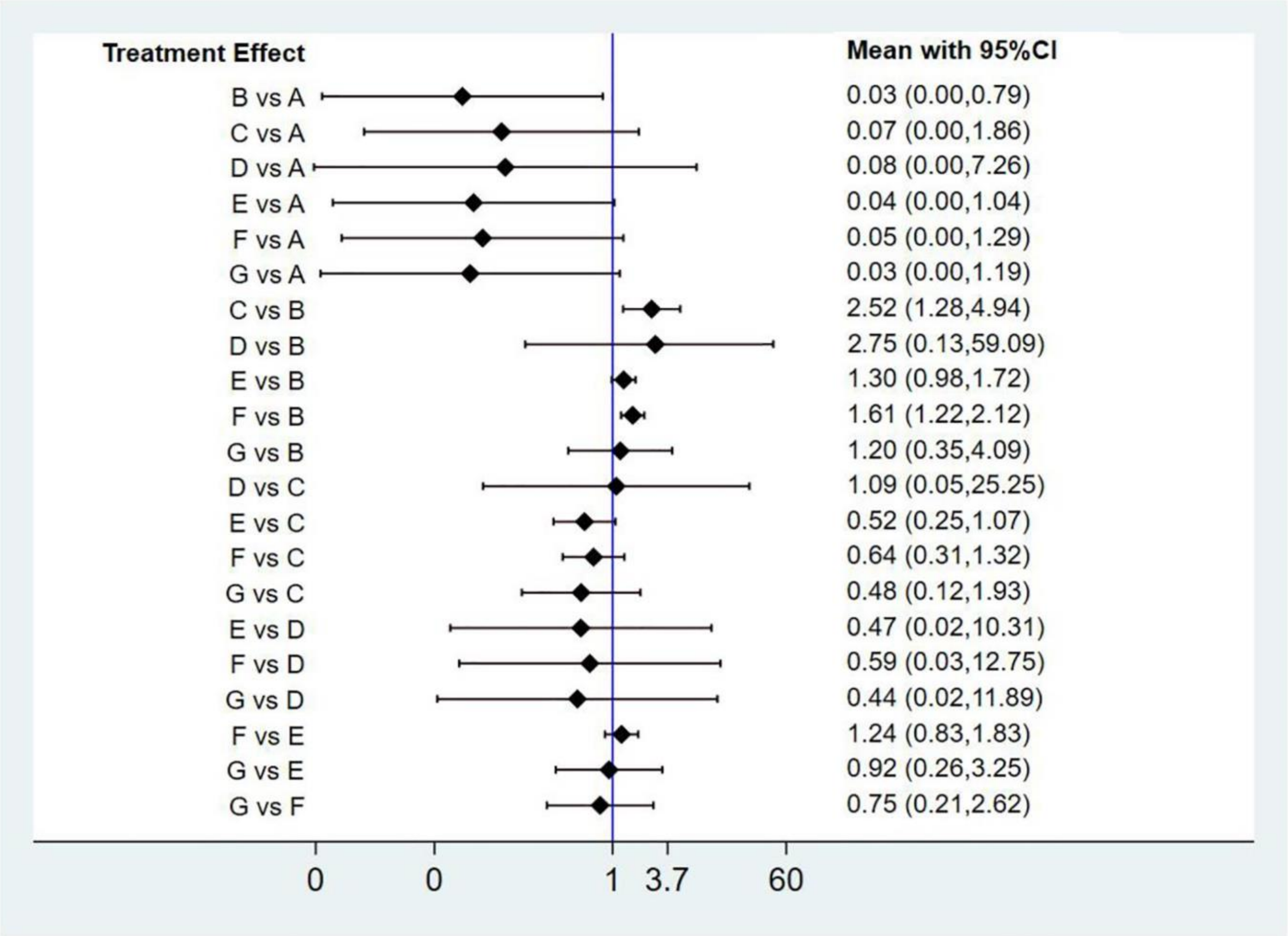

Supplementary Figure 13. Forest plots of thrombosis. Abbreviations: A: placebo/control; B: ESAs; C: daprodustat; D: molidustat; E: vadadustat; F: roxadustat; G: enarodustat.

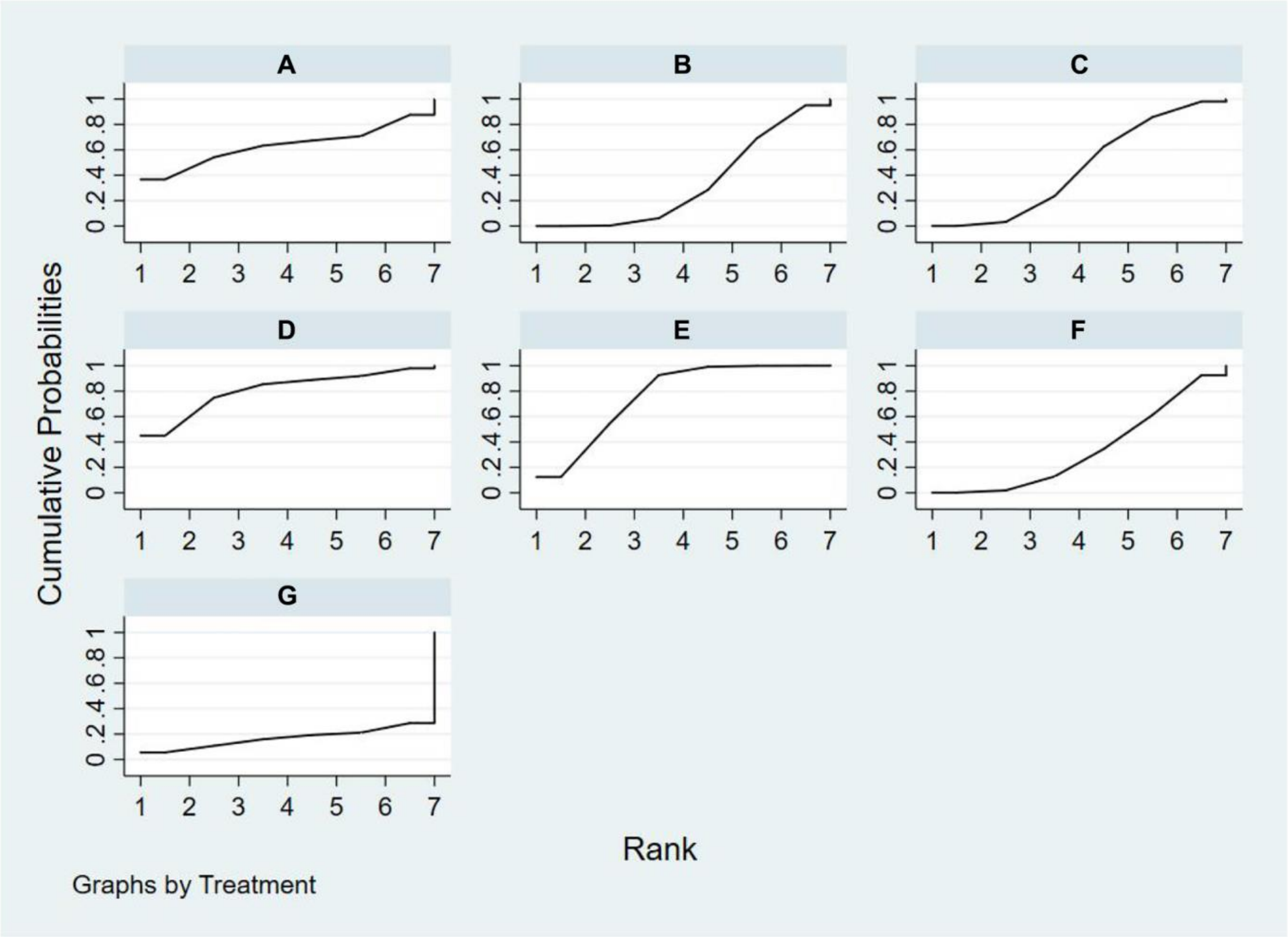

Supplementary Figure 14. The surface under the cumulative ranking curve for hypertension. (**A**) Placebo/control; (**B**) ESAs; (**C**) daprodustat; (**D**) molidustat; (**E**) vadadustat; (**F**) roxadustat; (**G**) enarodustat.

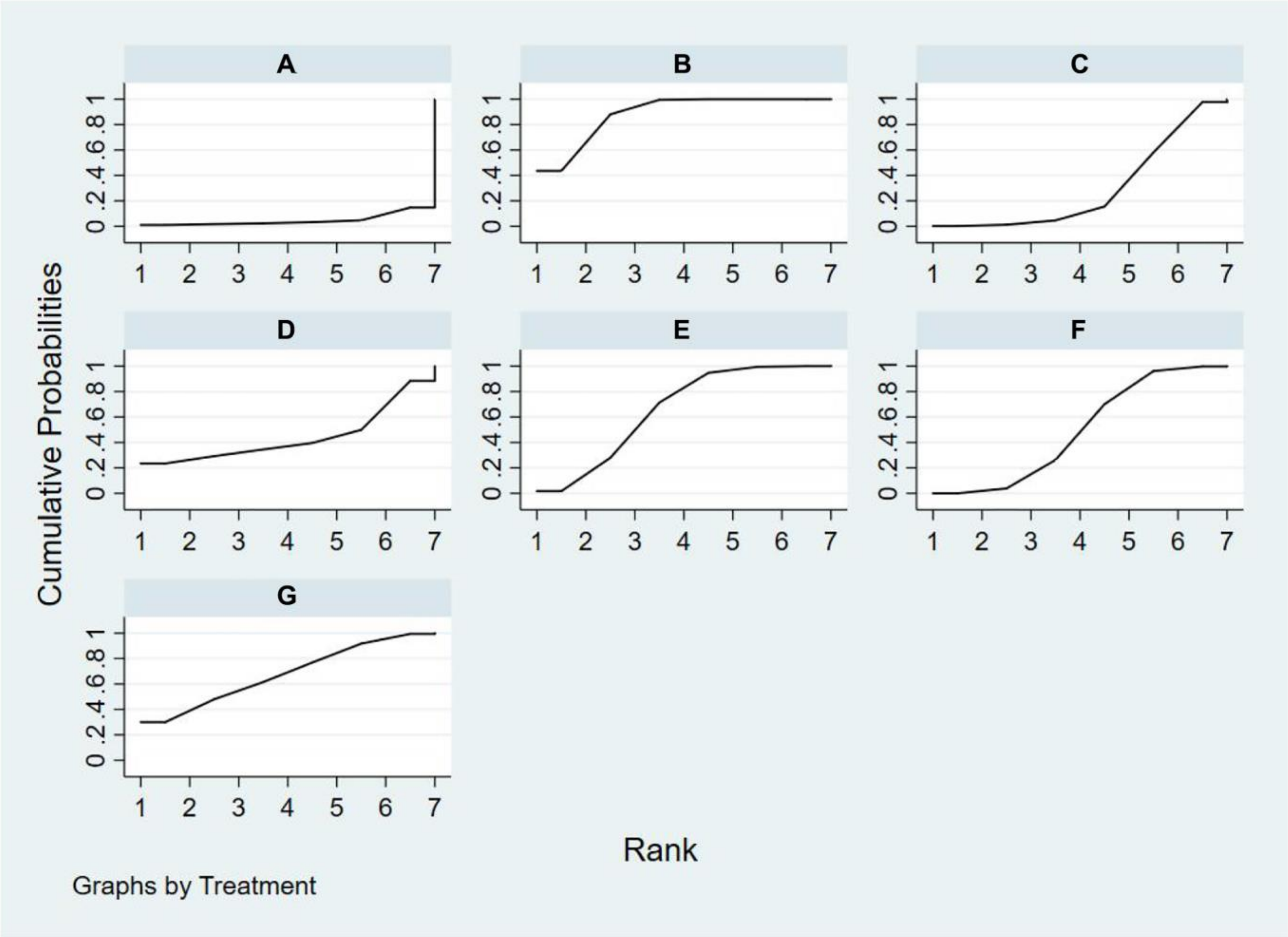

Supplementary Figure 15. The surface under the cumulative ranking curve for thrombosis. (**A**) Placebo/control; (**B**) ESAs; (**C**) daprodustat; (**D**) molidustat; (**E**) vadadustat; (**F**) roxadustat; (**G**) enarodustat.

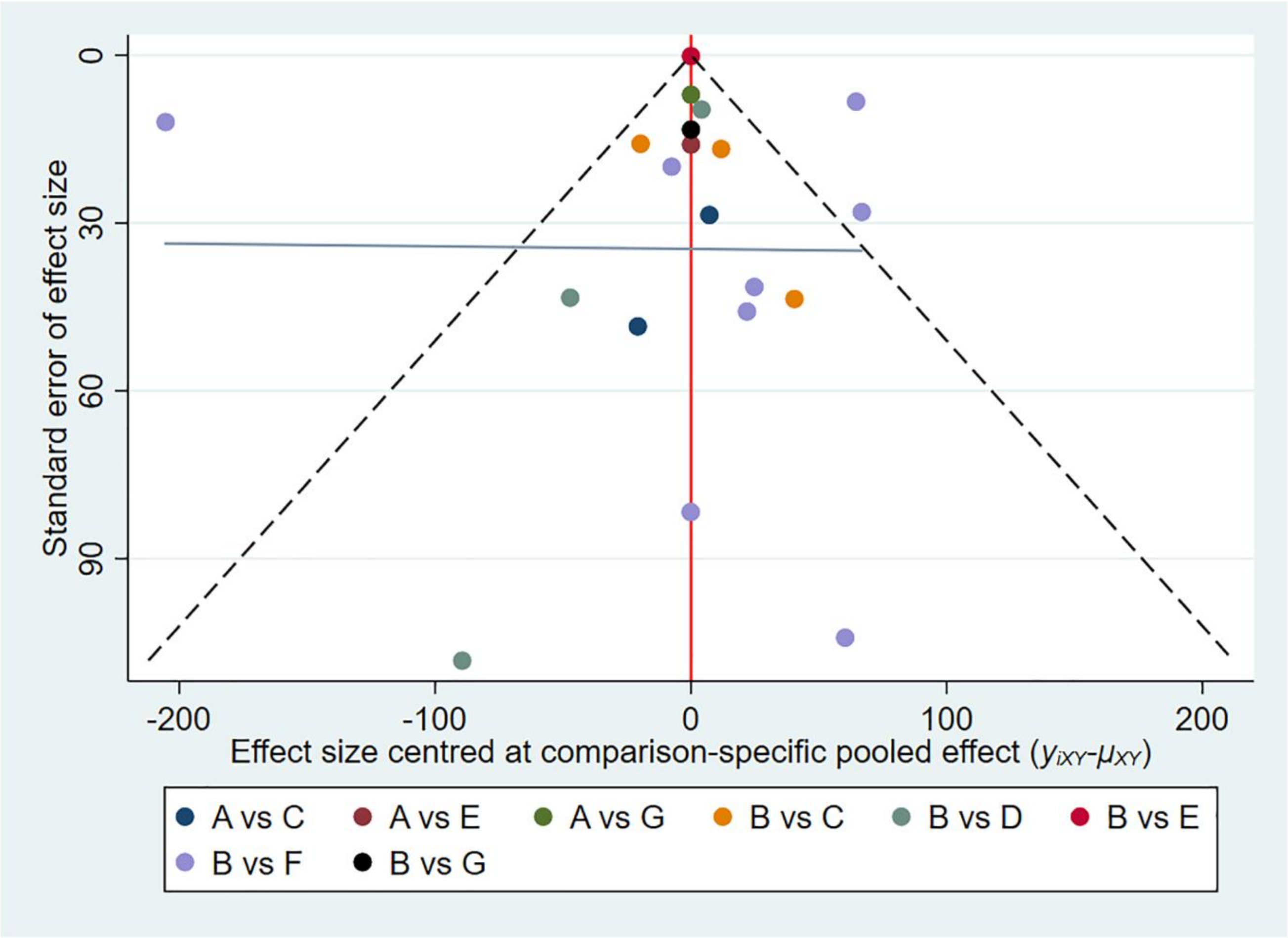

Supplementary Figure 16. Funnel plot assessing ferritin. Abbreviations: A: placebo/control; B: ESAs; C: daprodustat; D: molidustat; E: vadadustat; F: roxadustat; G: enarodustat.

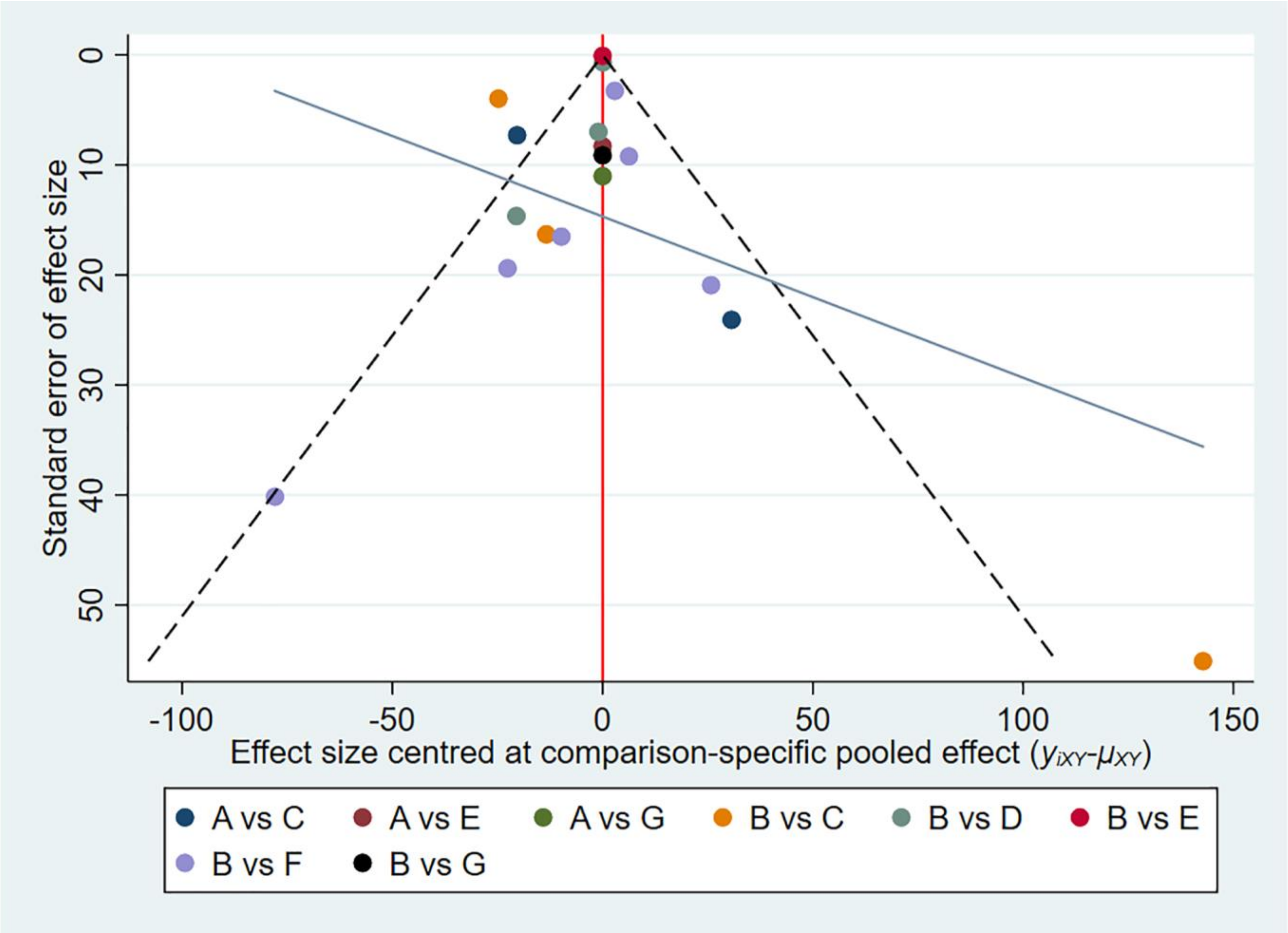

Supplementary Figure 17. Funnel plot evaluating hepcidin. Abbreviations: A: placebo/control; B: ESAs; C: daprodustat; D: molidustat; E: vadadustat; F: roxadustat; G: enarodustat.

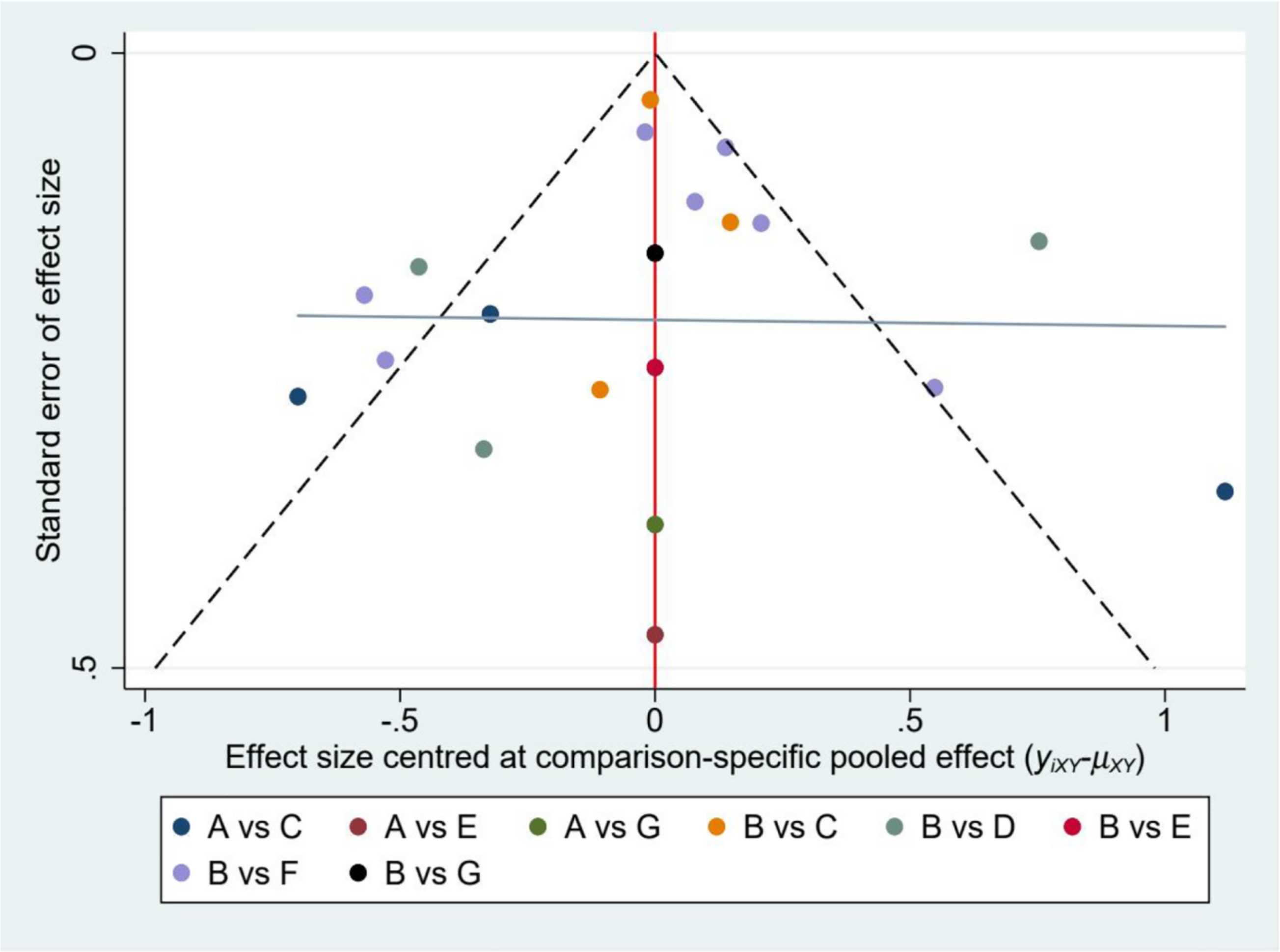

Supplementary Figure 18. Funnel plot evaluating TIBC. Abbreviations: A: placebo/control; B: ESAs; C: daprodustat; D: molidustat; E: vadadustat; F: roxadustat; G: enarodustat.

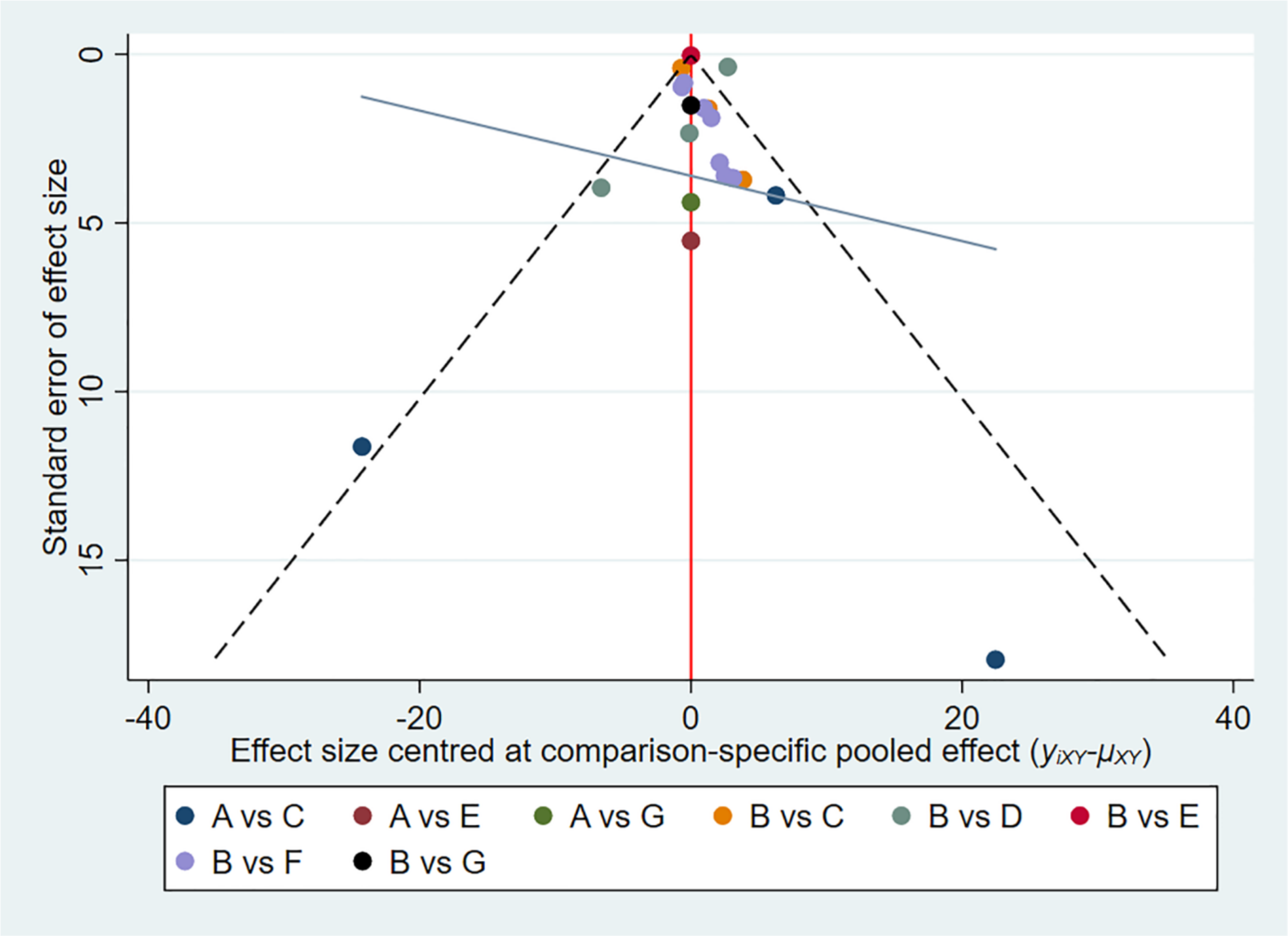

Supplementary Figure 19. Funnel plot evaluating TSAT. Abbreviations: A: placebo/control; B: ESAs; C: daprodustat; D: molidustat; E: vadadustat; F: roxadustat; G: enarodustat.

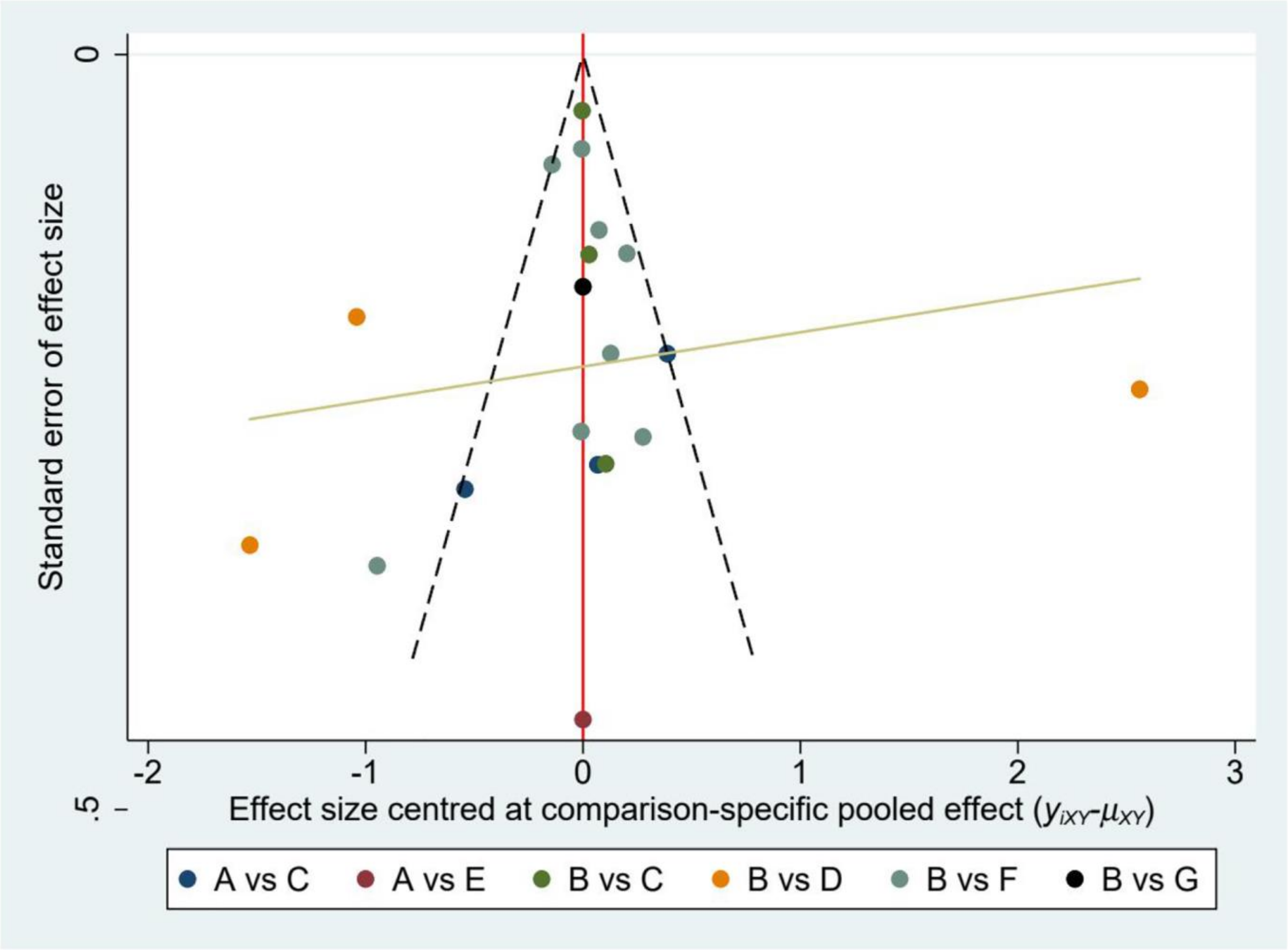

Supplementary Figure 20. Funnel plot evaluating serum iron. Abbreviations: A: placebo/control; B: ESAs; C: daprodustat; D: molidustat; E: vadadustat; F: roxadustat; G: enarodustat.

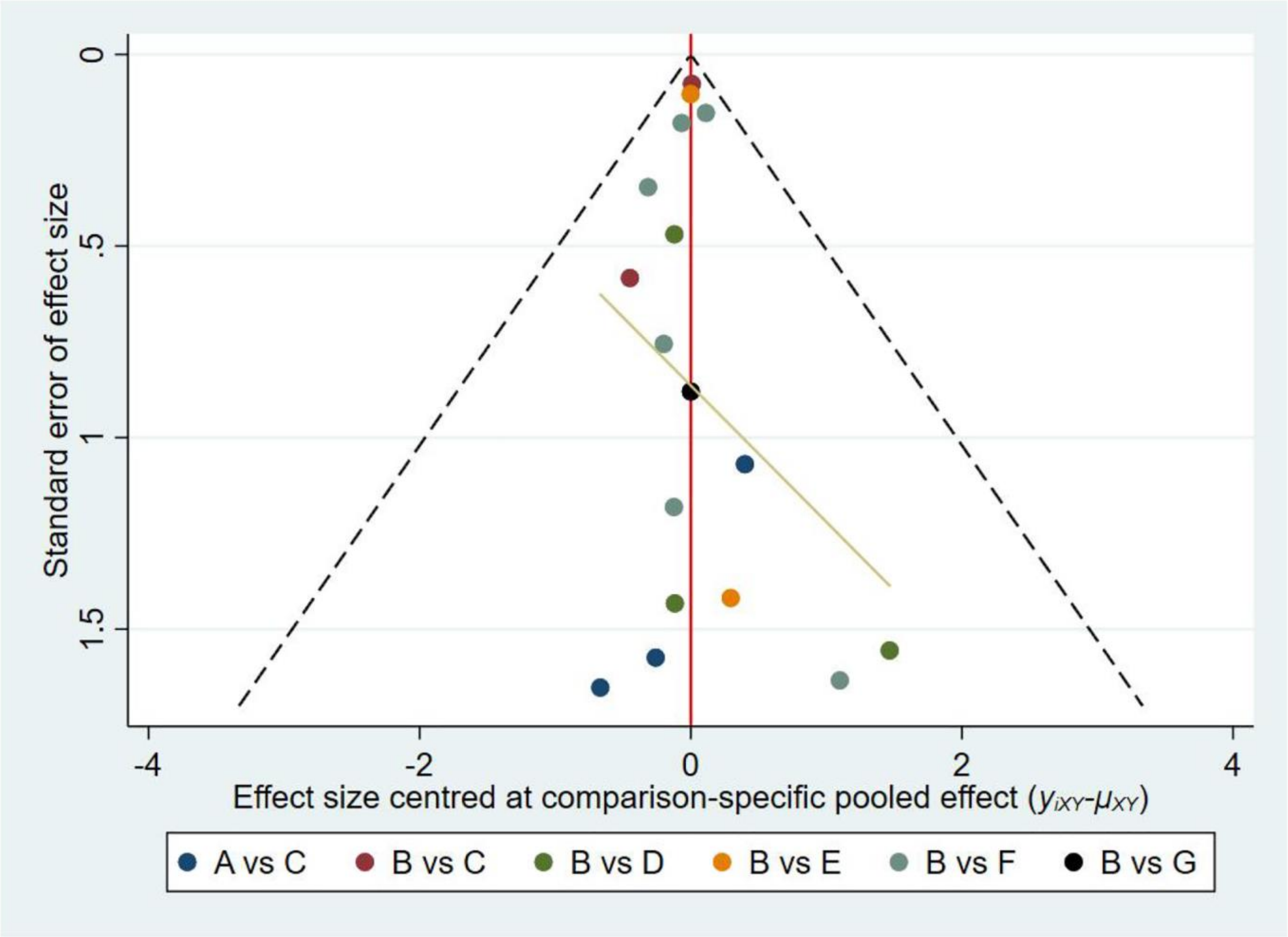

Supplementary Figure 21. Funnel plot evaluating hypertension. Abbreviations: A: placebo/control; B: ESAs; C: daprodustat; D: molidustat; E: vadadustat; F: roxadustat; G: enarodustat.

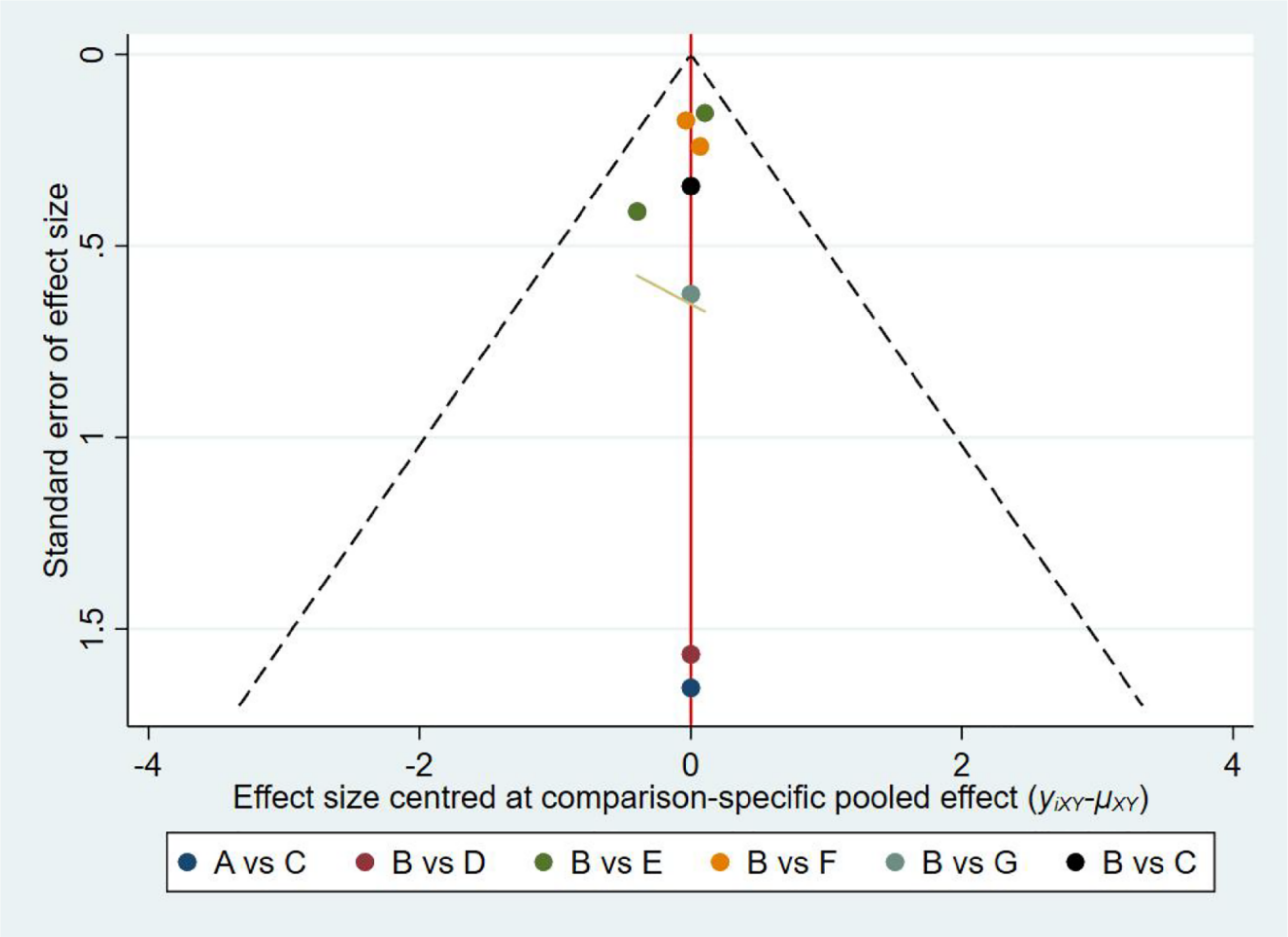

Supplementary Figure 22. Funnel plot evaluating thrombosis. Abbreviations: A: placebo/control; B: ESAs; C: daprodustat; D: molidustat; E: vadadustat; F: roxadustat; G: enarodustat.
