# Supplementary material for: A network meta-analysis of the efficacy of hypoxia-inducible factor prolyl-hydroxylase inhibitors in dialysis chronic kidney disease
Source: Aging (Albany NY). 2023 Mar 27;15(6):2237–74. doi: 10.18632/aging.204611 (PMC10085583; doi:10.18632/aging.204611)
Supplement: Supplementary Table 1 [file aging-15-204611-s002.pdf]

Supplementary Table 1. Baseline characteristics of the study populations.

Author /year	*Akizawa/2017*		*Akizawa/2020*		*Bailey/2019*		*Brigandi/2016*	
Characteristic	Daprodustat	Placebo	Daprodustat	DA	Daprodustat	Placebo	Daprodustat	Placebo
Number	78	19	136	135	79	18	31	6
Age- years±SD	62.1±9.7	63.4±9.0	64±10	64±11	64.1±15.0	60.9±13.3	50.1±10.9	57.2±7.0
BMI, kg/m^2	22±3	20±3	22±3.8	23±4.2	28.2±7.5	30.6±8.6	24±4	26±5
Men (%)	38 (48)	7 (37)	91 (67)	89 (66)	50 (63)	7 (39)	19 (61)	2 (33)
Double-blind	Yes		Yes		Yes		No	
Ferritin, µg/l	211.09±234.12	228.2±137.13	86.5 (108.9)	96.6 (124.5)	NA	NA	NA	NA
Transferrin, g/dl	1.68±0.31	1.6±0.29	NA	NA	1.6±0.30	1.6±0.36	NA	NA
TIBC,µmol/l	42.77±6.14	42.1+6.3	254 (39)	249 (38)	37.7±6.47	39.8±7.97	NA	NA
Serum iron, µmol/l	17.96±4.85	17.8±4.01	68 (25)	65 (23)	12.1±4.62	13.9±4.38	NA	NA
Hepcidin, µg/l	308.6(72.8,1081.7)	401.1(95.9,688.6)	54.1 (107.9)	60.1 (105.0)	NA	NA	NA	NA
TSAT, %	39.3(34.5,44.9)	40.8(36.9,45.1)	27.0 (10.1)	26.3 (8.9)	30.7 (35.66)	33.7(26.95)	NA	NA
Hgb, g/dl	9.77±0.48	18±9.92	10.9 (0.8)	10.8 (0.7)	10.6 ± 0.7	10.6 ± 0.8	NA	NA
Hypertension (%)	NA	NA	127 (93)	125 (93)	NA	NA	NA	NA
Diabetes (%)	NA	NA	56 (41)	52 (39)	NA	NA	NA	NA
Myocardial infarction (%)	NA	NA	NA	NA	NA	NA	NA	NA
Hemofiltration/Hemodiafiltration, n(%)	8(10)	4(21)	50 (37)	47 (35)	18 (21)	3 (16)	NA	NA
Hemodialysis, n(%)	69(88)	15 (79)	86 (63)	88 (65)	66 (79)	16 (84)	NA	NA
Peritoneal dialysis, n(%)	0(0)	0(0)	0(0)	0(0)	0(0)	0(0)	NA	NA

Author /year	*Holdstock/2016*		*Meadowcroft/2018*		*Singh/2021*		*Akizawa/2020*	
Characteristic	Daprodustat	rhEPO	Daprodustat	Control	Daprodusta	ESA	Roxadustat	DA
Number	62	20	171	39	1487	1477	150	151
Age- years±SD	57.8±16.8	64.2±12.8	59.6±13.3	59.7±18.7	58 (48-67)	58 (47-68)	64.6 (11.7)	64.9(10.1)
BMI, kg/m^2	30±8	29±8	27.7±7.5	27.2±5.8	26.8 (23.0-31.2)	26.8(23.2-31.3)	NA	NA
Men (%)	43 (69)	16 (80)	108 (63)	26 (67)	851 (57.2)	847 (57.3)	101 (67.3)	107 (70.9)
Double-blind	Yes		Yes		No		Yes	
Ferritin, µg/l	734.96±386.8	441.76±252.4	585.1±429.7	459.0±261.0	589 (344–976)	604 (341–948)	102.31(83.45)	96.28 (75.14)
Transferrin, g/dl	1.96±0.4	1.76±0.5	NA	NA	NA	NA	1.802(0.327)	1.810(0.297)
TIBC,µmol/l	44.66±8.5	41.16±8.9	41.6±6.6	41.2±7.2	39 (34–43)	39 (34– 43)	43.4 (7.0)	43.5 (6.2)
Serum iron, µmol/l	12.56±5.5	12.86±3.4	13.4±6.1	13.4±4.9	13 (10–16)	13 (10–16)	12.1 (5.1)	12.6 (4.5)
Hepcidin, µg/l	483.1(93.5,1241.8)	286.3(61.5,893.7)	388.2(352.9,427.1)	334.4(274.7,407.1)	172.7(109.3-256)	179.6(108.4–251.9)	NA	NA
TSAT, %	32.66±12.0	28.46±12.3	29.6(27.9, 31.5)	30.7(27.0, 35.0)	33 (26–41)	32 (26–42)	28.28 (11.70)	29.04 (10.18)
Hgb, g/dl	NA	NA	10.4 (0.66)	10.6 (0.94)	10.35±0.97	10.39±0.98	11.02 (0.56)	11.01 (0.60)
Hypertension, n (%)	19 (95)	20 (100)	160 (90)	37 (95)	1366 (91.9)	1373 (93.0)	NA	NA
Diabetes, n (%)	8 (40)	11 (55)	62 (35)	18 (46)	615 (41.4)	617 (41.8)	54 (36.0)	54 (35.8)
Myocardial infarction n(%)	NA	NA	NA	NA	122 (8.2)	135 (9.1)	NA	NA
Hemofiltration/Hemodiafiltration, n(%)	0(0)	0(0)	57(32)	7(18)	0(0)	0(0)	0(0)	0(0)
Hemodialysis, n(%)	62(100)	20(100)	120(68)	33(85)	1316 (88.5)	1308 (88.6)	150(100)	151(100)
Peritoneal dialysis, n(%)	0(0)	0(0)	0(0)	0(0)	171 (11.5)	169 (11.4)	0(0)	0(0)

Author /year	*Barratt/2021*		*Chen/2017*		*Chen/2019*		*Chen/2021*
Characteristic	Roxadustat	ESA	Roxadustat	rhEPO	Roxadustat	Epoetin alfa	All populations
Number	2354	2360	74	22	204	100	55
Age- years±SD	55.5±14.9	56.3±14.6	50.8±12.6	53.8±10.0	47.6±11.7	51.0±11.8	47.6 6 10.3
BMI, kg/m^2	NA	NA	NA	NA	NA	NA	25.0 6 3.5
Men (%)	1365 (58)	1379 (58.4)	45 (61)	13 (59.1)	126 (61.8)	58 (58)	26 (47.3)
Double-blind	No		No		No		No
Ferritin, µg/l	NA	NA	450.5(369.8)	458(361)	498.5±487.4	420.1±406.8	393.2±254.8
Transferrin, g/dl	NA	NA	189.3(45.5)	187(35)	1.89±0.46	1.91±0.39	1.7±0.5
TIBC,µmol/l	NA	NA	217.3(50.2)	214(38)	47.4±11.4	48.3±9.0	NA
Serum iron, µmol/l	NA	NA	71.5(32.9)	79(31.9)	NA	NA	12.2±7.0
Hepcidin, µg/l	NA	NA	176.3(120.6)	209(127.1)	NA	NA	NA
TSAT, %	NA	NA	31.56(16.91)	34.1(14.6)	33.8±16.6	30.0±13.8	NA
Hgb, g/dl	10.32 (0.99)	10.37 (0.99)	10.6(1.0)	10.8(0.7)	10.4±0.7	10.5±0.7	7.8±1.3
Hypertension, n (%)	684 (29.1)	707 (30.0)	NA	NA	NA	NA	18 (32.7)
Diabetes, n (%)	799 (33.9)	813 (34.4)	NA	NA	30 (14.7)	17 (17.0)	NA
Myocardial infarction n(%)	NA	NA	NA	NA	NA	NA	NA
Hemofiltration/Hemodiafiltration, n(%)	NA	NA	0(0)	0(0)	0(0)	0(0)	NA
Hemodialysis, n(%)	2137 (90.8)	2156 (91.4)	74(100)	22(100)	182 (89.2)	89 (89.0)	NA
Peritoneal dialysis, n(%)	0(0)	0(0)	0(0)	0(0)	22 (10.8)	11 (11.0)	NA

Author /year	*Csiky/2021*		*Hou/2021*		*Provenzano/2016*		*Provenzano/2021*	
Characteristic	Roxadustat	ESA	Roxadustat	ESAs	Roxadustat	Epoetin alfa	Roxadustat	Epoetin alfa
Number	414	420	86	43	67	23	522	521
Age- years±SD	61.0±13.8	61.8±13.4	48±12	48.3±13	56.9±12.1	57.0±11.6	53.8±14.7	54.3±14.6
BMI, kg/m2	26.87 (4.86)	26.95 (5.59)	23.7±3.5	24.1±3.2	NA	NA	NA	NA
Men (%)	245 (59.2)	235 (56)	47 (55)	25 (58)	45 (67)	14 (61)	309 (59.2)	307 (58.9)
Double-blind	No		Yes		No		No	
Ferritin, μg/l	NA	NA	268.8±297.2	257.4±190.8	827.7±474.3	1,065.8±657.2	441.4 (337.0)	437.4 (311.4)
Transferrin, g/dl	NA	NA	2.0±0.5	1.9±0.6	NA	NA	NA	NA
TIBC,μmol/l	NA	NA	42.5±11.7	41.3±14.9	199.7±34.0	202.1±26.7	241.04 (43.00)	238.06 (37.04)
Serum iron, μmol/l	NA	NA	12.6±5.0	11.4±5.0	66.4±20.6	63.3±32.0	64.41 (24.24)	65.52 (24.15)
Hepcidin, μg/l	NA	NA	NA	NA	327.1±178.8	298.7±123.1	173.21 (120.21)	169.91 (127.98)
TSAT, %	NA	NA	31.3±14.2	29.6±13.2	29.2±10.0	28.1±14.4	27.0 (9.3)	27.6 (8.9)
Hgb, g/dl	10.75 (0.62)	10.78 (0.62)	9.0±1.4	9.0±1.2	11.2±0.7	11.2±1.0	8.4 (1.0)	8.5 (1.0)
Hypertension, n (%)	124 (30.0)	120 (28.6)	29 (34)	14 (33)	66(100)	22(100)	505 (96.7)	504 (96.7)
Diabetes, n (%)	74 (17.9)	95 (22.6)	12 (14)	8 (19)	39(59)	14(64)	205 (39.3)	204 (39.2)
Myocardial infarction n(%)	NA	NA	NA	NA	NA	NA	33 (6.3)	33 (6.3)
Hemofiltration/Hemodiafiltration, n(%)	0(0)	0(0)	0(0)	0(0)	0(0)	0(0)	0(0)	0(0)
Hemodialysis, n(%)	379 (91.5)	405 (96.4)	0(0)	0(0)	67(100)	23(100)	469 (89.8)	462 (88.7)
Peritoneal dialysis, n(%)	35 (8.5)	15 (3.6)	86(100)	43(100)	0(0)	0(0)	53 (10.2)	58 (11.1)

Author /year	*Akizawa/2019*		*Akizawa/2019*		*Akizawa/2021*		*Macdougall/2018*		NCT02174731	
Characteristic	Molidustat	Control	Molidustat	Epoetin	Molidustat	DA	Molidustat	Epoetin	Roxadustat	Epoetin Alfa
Number	59	15	57	30	153	76	157	42	1051	1055
Age- years±SD	60.8±12.3	56.7±10.1	61±12	59±9	66.2±10.3	64.8±10.6	59±13	59±9	53.5 (15.30)	54.5 (14.97)
BMI, kg/m2	NA	NA	NA	NA	22.61 (3.34)	22.25 (3.12)	NA	NA		
Men (%)	32 (54)	7 (67)	33 (58)	23 (77)	91 (59.5)	49 (64.5)	91 (58)	29 (69)	625(59.5)	626(59.3)
Double-blind	No		No		Yes		Yes		Yes	
Ferritin, μg/l	559.9±357.9	528.1±319.7	NA	NA	118.7 (115.5)	115.4 (119.1)	557 (315)	542 (331)	NA	NA
Transferrin, g/dl	NA	NA	NA	NA	NA	NA	NA	NA	NA	NA
TIBC,μmol/l	36.3±5.5	36.1±7.1	NA	NA	42.1 (6.6)	42.1 (7.0)	36 (6)	35 (7)	NA	NA
Serum iron, μmol/l	69.4±29.5	63.3±20.3	NA	NA	67.6 (24.1)	70.1 (27.1)	68 (25)	64 (21)	NA	NA
Hepcidin, μg/l	76.1±46.8	76.2±42.1	NA	NA	48.0 (40.1)	48.9 (41.5)	72 (40)	70 (36)	NA	NA
TSAT, %	34.0±12.4	32.4±12.0	NA	NA	29.2 (11.1)	29.8 (10.0	34 (11)	33 (12)	NA	NA
Hgb, g/dl	10.5 (0.6)	10.6 (0.6)	NA	NA	10.77 (0.64)	10.84 (0.65)	10.5 (0.6)	10.6 (0.5)	NA	NA
Hypertension, n (%)	13 (22.0)	3 (20.0)	19 (33)	12 (40)	NA	NA	48 (31)	18 (43)	NA	NA
Diabetes, n (%)	22 (37.3)	7 (46.7)	30 (53)	18 (60)	48 (31.4)	24 (31.6)	86 (55)	24 (57)	NA	NA
Myocardial infarction n(%)	NA	NA	NA	NA	NA	NA	NA	NA	NA	NA
Hemofiltration/Hemodiafiltration, n(%)	0(0)	0(0)	NA	NA	NA	NA	NA	NA	NA	NA
Hemodialysis, n(%)	59(100)	15(100)	NA	NA	NA	NA	NA	NA	NA	NA
Peritoneal dialysis, n(%)	0(0)	0(0)	NA	NA	NA	NA	NA	NA	NA	NA

Author /year	*Akizawa/2019*		*Akizawa/2021*		*Eckardt/2021*		*Nangaku/2020*		*Nangaku/2021*	
Characteristic	Enarodusta	Placebo	Enarodusta	DA	Vadadustat	DA	Vadadusta	Placebo	Vadadustat	DA
Number	60	22	86	86	1777	1777	44	14	162	161
Age- years±SD	61.9 ± 11.2	60.7 ± 13.0	63.2 ± 10.8	64.8 ± 10.3	57.9±13.9	58.4±13.8	63.3±9.2	65.7±11.6	66.0±11.3	64.9±11.7
BMI, kg/m2	NA	NA	NA	NA	NA	NA	24±4.5	22.4±4	22.4±3.4	22.4±4.5
Men (%)	45 (75)	15 (68)	61 (70.9)	61 (70.9)	990 (55.7)	1004 (56.5)	32 (73)	8 (57)	104 (64.2)	109 (67.7)
Double-blind	Yes		Yes		No		Yes		Yes	
Ferritin, µg/l	98.6(36.7,169)	81.0(37.9, 115.0)	90.90(12.3,794)	90.30(12.4, 609)	NA	NA	217.8(113)	294.6(215)	144.5±139.6	140.0±95.3
Transferrin, g/dl	NA	NA	NA	NA	NA	NA	NA	NA	NA	NA
TIBC,µmol/l	231.5(207,250)	248.0(216.0,273)	233.0 (179, 341)	243.0 (172, 361)	NA	NA	233.3(35.6)	207.6(34.2)	NA	NA
Serum iron, µmol/l	63.5 (52.0, 77)	62.5 (52.0, 76)	65.5(33, 144)	64.0 (9, 167)	NA	NA	98.5 (31.5)	100.9 (25.5)	NA	NA
Hepcidin, µg/l	64.9(37.0,156)	67.1 (24.6, 127)	70.5(0.34,309)	51.5(1.67, 218)	NA	NA	140.1(61.3)	184.6 (94.3)	NA	NA
TSAT, %	27.5(23.3, 35.4)	23.7 (22.1, 30)	27.0(11, 69)	27.0 (4, 64)	NA	NA	43.63(16.6)	49.78 (15.4)	28.6±10.6	26.9±9.4
Hgb, g/dl	NA	NA	10.79 (0.65)	10.87 (0.70)	10.6±0.9	10.2±0.8	9.0 (0.6)	9.0 (0.6)	10.73±0.7	10.73±0.7
Hypertension, n (%)	NA	NA	NA	NA	NA	NA	13 (29.5)	6 (42.9)	152 (93.8)	147 (91.3)
Diabetes, n (%)	17(28.33)	2 (9.1)	31 (36.0)	36 (41.9)	971 (54.6)	998 (56.2)	19 (43)	5 (36)	35 (21.6)	49 (30.4)
Myocardial infarction n(%)	NA	NA	NA	NA	NA	NA	NA	NA	NA	NA
Hemofiltration/Hemodiafiltration, n(%)	NA	NA	0(0)	0(0)	NA	NA	NA	NA	NA	NA
Hemodialysis, n(%)	60(100)	22(100)	86(100)	86(100)	1652 (93.0)	1633 (91.9)	NA	NA	NA	NA
Peritoneal dialysis, n(%)	NA	NA	0(0)	0(0)	137 (7.7)	143 (8.0)	NA	NA	NA	NA

DA: darbepoetine; BMI: body mass index; TIBC: total iron-binding capacity; TSAT: transferrin saturation; Hgb: hemoglobin
