# Supplementary material for: A network meta-analysis of the efficacy of hypoxia-inducible factor prolyl-hydroxylase inhibitors in dialysis chronic kidney disease
Source: Aging (Albany NY). 2023 Mar 27;15(6):2237–74. doi: 10.18632/aging.204611 (PMC10085583; doi:10.18632/aging.204611)
Supplement: Supplementary Tables 2 and 3 [file aging-15-204611-s003.pdf]

SUPPLEMENTARY TABLES

Supplementary Table 2. Node-splitting approach for inconsistency assessment of all comparisons.

Side	Direct		Indirect		Difference		*P*
	Coef	Std	Coef	Std	Coef	Std	
A vs. C	1.07	0.21	1.54	0.34	−0.48	0.39	0.23
A vs. E	1.54	0.28	0.94	0.31	0.59	0.41	0.15
A vs. G	1.51	0.49	1.62	0.39	−0.11	0.62	0.86
B vs. C	−0.06	0.17	−0.54	0.36	0.48	0.39	0.23
B vs. D	−0.05	0.20	−2.68	314.9	2.62	314.9	0.99
B vs. E	−0.20	0.20	0.39	0.36	−0.59	0.41	0.15
B vs. F	0.32	0.11	−2.76	111.1	3.08	111.1	0.98
B vs. G	0.27	0.32	0.16	0.54	0.11	0.86	0.86

Abbreviations: Coef: coefficient; Std: standard deviation; A: placebo/control; B: ESAs; C: daprodustat; D: molidustat; E: vadadustat; F: roxadustat; G: enarodustat.

Supplementary Table 3. Ranking probabilities, mean ranks, and SUCRA values.

	placebo	ESAs	daprodustat	molidustat	vadadustat	roxadustat	enarodustat
Hemoglobin							
Best	0	0	0.2	3.1	1.7	57.4	37.5
2nd	0	7.1	2.6	11.3	8.7	36.9	33.4
3rd	0	33	9.1	20.7	20.1	5.1	12
SUCRA	0	53.9	33.7	46.4	44.8	91.8	79.4
PrBest	0	0	0.2	3.1	1.7	57.4	37.5
Mean Rank	7	3.8	5	4.2	4.3	1.5	2.2
Ferritin							
Best	0	0.3	10	6	11.5	63.9	8.3
2nd	0.1	13.6	23.6	11.6	15.7	22.8	12.6
3rd	0.6	30.4	21.9	10.9	14.2	9.2	12.8
SUCRA	6.5	54.7	60.9	40.3	51.0	90.9	45.8
PrBest	0	0.3	10.0	6.0	11.5	63.9	8.3
Mean Rank	6.6	3.7	3.3	4.6	3.9	1.5	4.3
Hepcidin							
Best	0	0	18.9	2.1	10.6	23.5	44.9
2nd	0	0.1	26	6.0	15.8	28.5	23.5
3rd	0	2.8	25.4	11.3	21.5	24.4	14.7
SUCRA	1.7	29.8	69.6	37.9	56.2	74	80.9
PrBest	0	0	18.9	2.1	10.6	23.5	44.9
Mean Rank	6.9	5.2	2.8	4.7	3.6	2.6	2.1
TSAT							
Best	0	17.2	6.1	0	0.5	6.7	69.5
2nd	0	55.5	13.2	0.3	2.4	17.8	10.8
3rd	0	21.8	22.1	2.2	8.5	37.3	8.1
SUCRA	0	80.7	56.3	20.6	39.9	63.7	88.7
PrBest	0	17.2	6.1	0	0.5	6.7	69.5
Mean Rank	7.0	2.2	3.6	5.8	4.6	3.2	1.7
TIBC							

Best	0	0	0	0	92.6	0	7.3
2nd	0	0	7.7	3.1	7.3	3.1	78.7
3rd	0.4	0	50.7	15.3	0.1	24.6	8.8
SUCRA	12.2	14.0	58.1	37.3	98.7	48.7	80.9
PrBest	0	0	0	0	92.6	0	7.3
Mean Rank	6.3	6.2	3.5	4.8	1.1	4.1	2.1
Serum iron							
Best	5.6	27.2	4.7	0.9	25.6	11.7	24.3
2nd	11.2	36.3	8.8	2.0	8.5	22.7	10.5
3rd	11.2	21.0	19.1	5.7	7.5	23.1	12.4
SUCRA	41.0	78.3	48.0	20.5	48.0	61.3	52.9
PrBest	5.6	27.2	4.7	0.9	25.6	11.7	24.3
Mean Rank	4.5	2.3	4.1	5.8	4.1	3.3	3.8
Hypertension							
Best	36.8	0	0.1	45	12.4	0.2	5.6
2nd	17.4	0.4	3.1	29.9	42.3	1.7	5.2
3rd	9.1	5.7	20.4	10.6	38	11.1	5.1
SUCRA	63.3	33.2	45.6	80.7	76.5	33.9	16.9
PrBest	36.8	0	0.1	45	12.4	0.2	5.6
Mean Rank	3.2	5.0	4.3	2.2	2.4	5.0	6.0
Thrombosis							
Best	1.0	43.6	0.2	23.5	1.7	0.0	30.0
2nd	0.7	44.4	1.0	5.8	26.3	3.8	18.0
3rd	0.7	11.4	3.4	5.2	43.4	22.4	13.5
SUCRA	4.6	88.5	29.6	44.2	65.9	49.3	67.9
PrBest	1.0	43.6	0.2	23.5	1.7	0.0	30.0
Mean Rank	6.7	1.7	5.2	4.4	3.0	4.0	2.9

Abbreviations: SUCRA: surface under the cumulative ranking curve; ESAs: erythropoiesis-stimulating agents; TSAT: transferrin saturation; TIBC: total iron-binding capacity.
